# Network approach identifies Pacer as an autophagy protein involved in ALS pathogenesis

**DOI:** 10.1186/s13024-019-0313-9

**Published:** 2019-03-27

**Authors:** S. Beltran, M. Nassif, E. Vicencio, J. Arcos, L. Labrador, B. I. Cortes, C. Cortez, C. A. Bergmann, S. Espinoza, M. F. Hernandez, J. M. Matamala, L. Bargsted, S. Matus, D. Rojas-Rivera, M. J. M. Bertrand, D. B. Medinas, C. Hetz, P. A. Manque, U. Woehlbier

**Affiliations:** 1 Center for Integrative Biology, Faculty of Science, Universidad Mayor, Camino la Piramide 5750, P.O.BOX 70086 Santiago, Chile; 2 Center for Genomics and Bioinformatics, Faculty of Science, Universidad Mayor, Camino la Piramide, 5750 Santiago, Chile; 3 Department of Neurological Sciences, Faculty of Medicine, University of Chile, Santiago, Chile; 4 Biomedical Neuroscience Institute, Faculty of Medicine, University of Chile, Independencia, 1027 Santiago, Chile; 5 Fundación Ciencia & Vida, Zañartu 1482, 7780272 Santiago, Chile; 6 Neurounion Biomedical Foundation, 7780272 Santiago, Chile; 7 Center for Geroscience, Brain Health and Metabolism (GERO), Santiago, Chile; 8 VIB Center for Inflammation Research, Technologiepark 927, Zwijnaarde, 9052 Ghent, Belgium; 9 Department of Biomedical Molecular Biology, Ghent University, Technologiepark 927, Zwijnaarde, 9052 Ghent, Belgium; 10 Buck Institute for Research on Aging, Novato, CA 94945 USA; 11 Program of Cellular and Molecular Biology, Institute of Biomedical Sciences, University of Chile, Independencia, 1027 Santiago, Chile; 12 Department of Immunology and Infectious Diseases, Harvard School of Public Health, Boston, MA 02115 USA; 13 Center for the Study of Biological Complexity, Virginia Commonwealth University, Richmond, VA 23298 USA

**Keywords:** ALS, Autophagy, Beclin1, C13orf18, KIAA0226-like, Pacer, Rubicon, Rubicon-like, SOD1, TDP43

## Abstract

**Background:**

Amyotrophic lateral sclerosis (ALS) is a multifactorial fatal motoneuron disease without a cure. Ten percent of ALS cases can be pointed to a clear genetic cause, while the remaining 90% is classified as sporadic. Our study was aimed to uncover new connections within the ALS network through a bioinformatic approach, by which we identified C13orf18, recently named Pacer, as a new component of the autophagic machinery and potentially involved in ALS pathogenesis.

**Methods:**

Initially, we identified Pacer using a network-based bioinformatic analysis. Expression of Pacer was then investigated in vivo using spinal cord tissue from two ALS mouse models (SOD1^G93A^ and TDP43^A315T^) and sporadic ALS patients. Mechanistic studies were performed in cell culture using the mouse motoneuron cell line NSC34. Loss of function of Pacer was achieved by knockdown using short-hairpin constructs. The effect of Pacer repression was investigated in the context of autophagy, SOD1 aggregation, and neuronal death.

**Results:**

Using an unbiased network-based approach, we integrated all available ALS data to identify new functional interactions involved in ALS pathogenesis. We found that Pacer associates to an ALS-specific subnetwork composed of components of the autophagy pathway, one of the main cellular processes affected in the disease. Interestingly, we found that Pacer levels are significantly reduced in spinal cord tissue from sporadic ALS patients and in tissues from two ALS mouse models. In vitro, Pacer deficiency lead to impaired autophagy and accumulation of ALS-associated protein aggregates, which correlated with the induction of cell death.

**Conclusions:**

This study, therefore, identifies Pacer as a new regulator of proteostasis associated with ALS pathology.

**Electronic supplementary material:**

The online version of this article (10.1186/s13024-019-0313-9) contains supplementary material, which is available to authorized users.

## Background

Amyotrophic lateral sclerosis (ALS) is the most common adult motoneuron disease that causes a progressive paralysis due to the selective loss of motoneurons in the motor cortex, brainstem motor nucleus, and spinal cord [1]. In recent years, a growing number of genetic loci have been associated with ALS and other complex diseases (reviewed in [2, 3]). The first mutations identified as a cause of familial ALS (fALS) mapped to *SOD1* (superoxide dismutase 1), whereas mutations in the *TARDBP* gene, coding for Tar DNA binding protein 43 (TDP43), one of the most common components of protein aggregates in ALS cases, were identified years later [4]. Since then, the number of genes associated with ALS has significantly increased (reviewed in [2]). The recent discovery of hexanucleotide G_4_C_2_ repeat expansions in the intronic region of C9orf72 as a common genetic cause of fALS [5] and frontotemporal dementia (FTD) has profoundly changed our understanding of ALS, explaining almost 40% of the familial cases, in addition to near 10% of sporadic ALS (sALS) [5, 6]. ALS is now considered to be part of a spectrum of neurological disorders instead of simply a neuromuscular disease. Understanding shared pathophysiological mechanisms of sALS and fALS promises the finding of effective therapies for both forms of the disease. Defective cellular processes identified thus far to be responsible for the ALS and the related FTD include (i) alterations to proteostasis control (protein quality control, including proteasomal degradation, autophagy and endoplasmic reticulum (ER) stress), (ii) mitochondrial dysfunction, (iii) cytoskeletal dynamics and axonal transport, (iv) RNA homeostasis, and (v) DNA damage response (reviewed in [3, 7, 8]). Macroautophagy, here referred to as autophagy, is an evolutionarily conserved process that consists of vesicles termed autophagosomes to deliver intracellular cargo to the lysosome, including long-lived cytosolic proteins, damaged organelles, and protein aggregates [9, 10]. Most cell types, including neurons, operate under constitutive autophagy [11], which is thought to have a vital role in maintaining their metabolic and proteostatic balance [12–14]. In this context, defects in the endolysosomal pathway or autophagy-related genes have been associated with diverse neurodegenerative diseases, including Alzheimer’s disease (AD), Huntington’s disease (HD), Parkinson’s disease (PD), as well as ALS [15, 16]. Mutations in several genes coding for proteins involved in autophagy or other membrane trafficking pathways have been found in ALS patients, including *SQSTM1* and *OPTN*, which encode the selective autophagy receptors SQSTM1/p62 and optineurin, respectively, and *ALS2* that encodes alsin2, which participates in membrane trafficking, as well as *TBK1* (TANK-binding kinase 1)*,* a kinase involved in autophagy-mediated degradation of ubiquitinated cargos [17–19]. *C9orf72* was also shown to participate in the autophagy process [20]. Targeting autophagy with genetic and pharmacological approaches has indicated that the pathway may have pathogenic or protective roles depending on the disease stage and cell type analyzed [21–23]. Thus, defects in autophagy and endolysosomal pathways may underlay an important part of the etiology of the disease.

The rise of high-throughput experimental approaches such as genomics, transcriptomics, and proteomics provides new datasets for ALS and other neurodegenerative diseases. Recent comprehensive studies proofed that network-based analysis offers the means to unravel new genes, pathways and disease networks associated with complex disorders similar to ALS [24–29]. The convergent analysis approach, which is loosely defined as the combination of multi-dimensional datasets with network modeling of gene and protein interactions, provides a new way to identify genes involved in disease pathways or mechanisms even with little or no previous evidence [26, 27]. We applied this approach to the data available for ALS and selected the *C13orf18* gene for further experimental studies, based on (i) its suggested participation in the human autophagy network through interaction with Beclin1, one of the main components of this pathway [30] that is known to be dysregulated in ALS [22], (ii) a single-nucleotide polymorphism (SNP) in the 3’UTR of the *C13orf18* gene (polymorphism ID: rs2478046) found in a higher allele frequency in a sALS patient cohort [31], and (iii) the fact that the C13orf18 protein was uncharacterized and had no cellular function assigned to it. Due to its sequence homology with Rubicon (Run domain protein Beclin1 interacting and cysteine-rich domain or KIAA0226), C13orf18 is also referred to as Rubicon-like or KIAA0226-L. However, recently, Cheng et al. reported that C13orf18 positively regulates autophagosome maturation by complex association with UVRAG and stimulation of Vps34 kinase activity, hence it was named Pacer (protein associated with UVRAG as autophagy enhancer) [32].

Here we report that Pacer displays decreased expression in the terminal stage of the disease in two fALS mouse models and notably in post-mortem spinal cord tissue from sALS patients. Interestingly, we found Pacer to be expressed exclusively in neurons in the spinal cord. Using cellular models, we demonstrate that targeting Pacer results in reduced autophagy activity, augmented SOD1 aggregation, and sensitization of motoneurons to cell death. Hence, our study identifies Pacer as a new protein involved in neuronal autophagy, whose loss is related to the selective vulnerability of motoneurons during ALS pathogenesis.

## Methods

### Convergent analysis

Copy number variation (CNV) data were collected from 4 published studies [33–36]. A total of 338 genes associated with ALS were included in the analysis. Additionally, genes linked to ALS were collected from The Huge Navigator (an integrated knowledge base of human genome epidemiology). We specifically searched the Phenopedia page for ALS related association studies and reported genes. Using the exact match of term “ALS” or “motoneuron disease,” we retrieved 143 genes annotated in the HuGE database (2011). From those, 128 genes were finally included in the analysis (14 genes were excluded from the analysis because there were not associated with the disease upon revision) (Additional file 1: Table S1). We uploaded the selected CNV and HuGE genes into the Ingenuity Pathway Analysis (IPA) system (Qiagen), which contains protein/protein interaction (PPI) and expression datasets. We ran a “core analysis” approach and obtained 241 genes in 12 ALS-associated subnetworks (Additional file 2: Table S2).

### Animals

We employed the SOD1^G93A^ (strain 002726) and TDP43^A315T^ (strain 010700) transgenic mice both obtained from The Jackson Laboratory as ALS mouse models [37, 38]. The TDP43^A315T^ received jellified food to prevent the premature death by intestinal dysmotility and allowed the motor degeneration phenotype to develop [39]. The animal care and all animal experiments were performed according to procedures approved by “Guide for the Care and Use of Laboratory Animals” (Commission on Life Sciences, National Research Council. National Academy Press 1996) and approved by the Bioethical Committee of the Universidad Mayor and from Neurounion Biomedical Foundation (Protocol CBA #05010).

### Histology and immunostaining

Mice were sacrificed and perfused with PBS, and spinal cords were collected. Spinal cords were fixed in paraformaldehyde (PFA, Merck, 30,525–89-4) 4% for 24 h and then paraffin embedded. The spinal cords were sectioned transversely in the lumbar section on a microtome at 10 μm and were collected in positively charged slides. Then, the slides were deparaffinized with xylol and hydrated with descending concentrations of alcohol until reaching distilled water. The epitopes were exposed with citrate buffer at 96 °C for 40 min. After this, slides were washed three times with 0.05% Tween20 in PBS. The blocking of non-specific binding sites was done with 3% BSA in PBS solution for 40 min at room temperature. Slides were incubated with primary antibodies diluted in 1% BSA-0.05% Tween20 in PBS solution overnight at 4 °C. Primary antibodies and dilutions were as follows: mouse anti-Pacer (custom-made from Abmart), 1:100, rabbit anti-Rubicon (Invitrogen, PA5–38017), 1:100, rabbit anti-GFAP (EMD Millipore Corp., AB5804), 1:1000, rabbit anti-NeuN (EMD Millipore Corp., ABN78), 1:1000 and anti-MMP9 (Abcam, ab38898). Secondary antibodies and dilutions were: anti-mouse Alexa 555 (Thermo Fisher Scientific, A28180), 1:1000 and anti-rabbit Alexa 488 (Thermo Fisher Scientific, A27034), 1:1000 prepared in a 1% BSA-0.05% Tween20 in PBS solution with DAPI (Invitrogen, D1306), 1:1000 or Hoechst (Life Technologies, H3570), 1:1000 incubated for 2 h. Then, slides were washed and mounted with Fluoromount-G (Invitrogen). Images were taken with a Leica TCS SP8 confocal microscope with a 10X, 40X and 63X objective magnifications. ImageJ and LAS X software were used to process the stacked images.

### Processing of human tissues

Frozen tissue was a generous gift from Dr. Robert H. Brown, Jr. (University of Massachusetts Medical School). Briefly, frozen post-­mortem spinal cord tissue from sALS patients and control subjects were obtained from the Alzheimer Disease Research Center at Massachusetts General Hospital and Dr. Robert H. Brown laboratory at the Neurology Department of University of Massachusetts Medical School under approved Institutional Review Board protocol (FWA#00004009). The biochemical analysis of the human samples was also authorized by the Ethics Committee of the Faculty of Medicine of the University of Chile.

### RNA extraction and quantitative real-time PCR

Total RNA was extracted from mouse tissue (liver, muscle, cortex, cerebellum, hippocampus, spinal cord) by first homogenizing tissues in saline phosphate buffer (100 μl of PBS, pH 7.4) with protease inhibitors (Thermo Fisher Scientific, A32955 in a weight per volume ratio of 1:1. The same protocol was followed for post-mortem human spinal cord samples preparation. 1 ml of TRIzol LS (Invitrogen, 10,296,028) was added to 50 μl of homogenate. The remaining 50 μl were kept for Western blot assays (see below). For RNA extraction from the mouse motoneuron cell line NSC34 (Neuroblastoma Spinal Cord 34) cells were pelleted, washed once with cold PBS and directly resuspended in 700 μl of TRIzol LS. Total RNA was isolated using the protocol recommended by the manufacturer. cDNAs were synthesized using the First Strand cDNA Synthesis Kit (Thermo Fisher Scientific, K1621). qRT-PCR was carried out via SYBR Green assays (Kappa biosystem, KK4601) using the Eco™ Real-Time PCR System (Illumina) according to the manufacturer’s instructions. Each sample was run in triplicates. mRNA expression was normalized to the 18 s (cells) or actin (tissues) rRNA expression. Transcript levels were quantified by using the ΔΔCt value method. The following primers were used for quantitative RT-PCR: *Pacer* forward 5′-TTCACCCACCAATCAAGAGGGACA-3 and reverse 5′-ACAAGACTCTGCAGATGAGTGGCA-3′; *Beclin1* forward 5′-CAGGAACTCACAGCTCCATTAC-3′ and reverse 5′-CCATCCTGGCGAGTTTCAATA-3′; *18S* forward 5′-GTAACCCGTTGAACCCCATT-3′ and reverse 5′-CCATCCAATCGGTAGTAGGG-3′, *Rubicon* forward 5′- TTCAGCATCTCCGAGTCCTT-3′ and reverse 5′- AATCCCGTGAACTGAACTGG-3′. For human samples, the primers used were: *hPacer* forward 5′-ATGGTGTCACAATCTACAGTCAGg-3′, reverse 5′-GGGAGAGGCAGCATCTGTC-3′; *hRubicon* forward 5′- CTGGCAGTTCGTGAAAGACA -3′ and reverse 5′- TTAGCAGGAAGGCAGCATCT-3′. *β-actin* forward 5′-AAGATCATTGCTCCTCCTGA-3′ and reverse 5′-TACTCCTGCTTGCTGATCCA-3′ primers were used for both mouse and human samples. RT-PCR conditions were: 1 cycle at 95 °C for 5 min, followed by 40 cycles at 95 °C for 30 s, 58 °C for 45 s.

### Plasmid constructs

To generate the plasmid vector encoding mPacer-V5 mouse *Pacer* mRNA was extracted from C57BL/6 spinal cord tissue and cloned into the pcDNA3.1/V5-His TOPO vector (Invitrogen, 460,083). The PCR was carried out in a final volume of 30 μl, containing 0.8 Units of DreamTaq (Thermo Fisher Scientific, EP0701), 3 μl of Dreamtaq Buffer (5x, Thermo Fisher, K1071), 0.6 μl of dNTPs (10 μM) and 0.45 μl (10 μM) of each primer, *Pacer* forward 5′- ATGAATTCAAGAGTCACGCCCAG -3′ and reverse 5′-TGTTGCTGCAGTGGGCAA -3′. PCR conditions were: 1 cycle at 95 °C for 5 min, followed by 35 cycles at 95 °C for 30 s, 55 °C for 30 s, and 72 °C for 2 min. The final extension step was carried out at 72 °C for 10 min. The PCR products were analyzed on 1% agarose gel. The ligation of the PCR product into the pcDNA3.1/V5-His TOPO vector was performed according to the manufacturer’s recommendation. The resulting pcDNA3.1/mPacer-V5-His plasmid was amplified in *E. coli DH5α,* and the correct insertion of the *Pacer* gene was verified by sequencing. The vector encoding hPacer-V5 was designed using the VectorBuilder online tool (Cyagen) and subsequently completely synthesized by and purchased from Cyagen. Plasmid for FLAG-tagged Beclin1 were kindly provided by Beth Levine [40].

### Cell culture, transfection, and viral transduction

We used HEK293T cells for immunoprecipitation assays and the NSC34 cell line as a motoneuron-cell like a model [41] for autophagy experiments, subcellular localization studies and mutant SOD1 aggregation and cell death analysis. NSC34 and HEK 293 T cells were grown in Dulbecco’s modified Eagle’s medium (DMEM, Gibco, 12,800,017) supplemented with 10% fetal bovine serum (FBS, Gibco, 10,438,026) and 1% penicillin/streptomycin (Biological Industries, DW1012), in a 5% CO_2_ incubator at 37 °C. All transfections were performed using the Effectene reagent (Qiagen, 301,427) according to the manufacturer’s recommendations. Plasmid DNAs were prepared using the Qiagen plasmid midi kit (Qiagen, 12,143) or the Axygen miniprep kit (Axygen, AP-MN-P-250). For viral transductions, lentivirus production was performed with Lenti-vpak (Origene, TR30037), according to the manufacturer’s recommendation. Briefly, HEK293T cells were transfected with scramble control (*shCtrl*) and shPacer constructs. Medium was replaced with fresh DMEM after 12 h, and viral supernatant was collected after 36 h and again after another 24 h. NSC34 cells were transduced with 2 ml viral supernatant. Stable NSC34 *shCtrl* and *shPacer* cell lines were established with Puromycin (Sigma, P8833-25 mg) (10 μg/ml) selection.

### Autophagy analysis

NSC34 cells were transfected with vectors encoding mouse Pacer-V5 or empty vector (pcDNA3.1/V5) (Invitrogen) or with vectors targeting mouse *Pacer* mRNA, *shPacer A* (GGACACAGAAGACGCCAGCAGGTTACGTG), *shPacer B* (TGGTACAACGGCCATTGGAGAGCTGTGTT) or a scramble control vector (*shCtrl)* (OriGene). To induce autophagy cells were treated with rapamycin (200 nM, Enzo Life Sciences, BML-A275) for 6 h. For the autophagy flux, cells were washed once with PBS and maintained with EBSS (Gibco, 24,010,043) medium for 0.5, 2, or 4 h. To inhibit autophagosome-lysosome fusion, cells were treated with a mix of bafilomycin A_1_ (0.5 μM), and protease inhibitors pepstatin (10 μg/ml), and E64D (10 μg/ml) for 4 h, all purchased from Merck.

### Immunofluorescence assay

NSC34 cells were seeded on coverslips and grown overnight. Briefly, cells were transfected and after 24 h cells washed in PBS, fixed with 4% paraformaldehyde (Merck) for 10 min at room temperature, permeabilized with 0.1% Triton X-100 (Merck, 9036-19-5) and 5% gelatin from cold water fish (Sigma, 9000-70-8) for 2 h at room temperature in a moisture chamber. Antibodies and concentrations employed were: mouse Pacer (custom-made from Abmart), 1:100; rabbit anti-Beclin1 (Santa Cruz Biotechnology, sc-11,427), 1:100. Secondary antibodies were used as follows: anti-mouse Alexa 555 (Thermo Fisher Scientific, A28180), 1:1000 and anti-rabbit Alexa 488 (Thermo Fisher Scientific, A27034), 1:1000. Nuclei were stained with Hoechst 33342 (Life Technologies, H3570)1:1000. Coverslips were mounted with Fluoromount G. Fixed cells were imaged with a Leica TCS SP8 confocal microscope. ImageJ and LAS X were employed to process the stacked images. The co-localization analysis was performed as in [42]. Briefly, confocal images were processed using CDA software. Applying the same thresholds for all experimental conditions, the software provides a coefficient of colocalization (Pearson’s correlation coefficient), which was used comparatively.

### Immunoprecipitation assay

HEK293T cells were co-transfected with vectors encoding mouse Pacer-V5 and Flag-tagged Beclin1. 48 h later, cell extracts were collected, centrifuged and resuspended in 500 μl of lysis buffer (0.2% NP40, 100 mM KCl, 50 mM Tris at pH 7.5, 150 mM NaCl, 250 mM PMSF) containing protease inhibitor cocktail 1 X (Thermo Fisher Scientific, A32955). After incubation overnight on ice, total cell extracts were subjected to immunoprecipitation (IP) with anti-V5 antibody conjugated-agarose beads (MBL International, 3315) for 2 h at 4 °C on a wheel rotor. Subsequently, beads were washed twice with lysis buffer and once with lysis buffer containing 500 mM NaCl. Protein complexes were eluted with V5 peptide. The input and IP eluate were separated by SDS-PAGE and assessed by Western blot analysis.

### Western blot analysis

Cells and tissues were homogenized in Triton buffer (1.0% Triton in PBS) containing protease inhibitor cocktail 1X by sonication. Protein concentration was determined by BCA assay (Pierce Thermo Fisher Scientific, 23,225). Antibody and dilutions used were: mouse anti-human-Pacer (Novus, B01P), 1:1000, rabbit anti-Rubicon (Cell Signaling, 8465), 1:1000, rabbit anti-LC3B (Cell Signaling Technology, 2575), 1:1000, mouse anti-SQSTM1/p62 (Abcam, ab56416), 1:10000, mouse anti-V5 (Thermo Fisher Scientific, R960-CUS), 1:4000, rabbit anti-Beclin1 (Santa Cruz Biotechnology, sc-11,427),1:1000, mouse anti-GFP (Santa Cruz Biotechnology, sc-9996), 1:2000, sheep anti-SOD1 (Calbiochem, 574,597), 1:1000, and custom mouse anti-Pacer antibody manufactured by Abmart raised against a 14 aa peptide of the N-terminal domain of mouse Pacer, 1:1000. Rabbit anti-HSP90 (Santa Cruz Biotechnologies, sc-7947) or rabbit anti-β-Actin (Cell Signaling Technology, 4967) were used as loading controls, 1:3000 or 1:1000, respectively. Secondary HRP-conjugated anti-rabbit (Life Technologies, 656,120), anti-mouse (Life Technologies, 626,520) or anti-sheep (Sigma-Aldrich, A3415) antibodies were employed at a 1:3000 dilution.

### Analysis of SOD1 aggregation

NSC34 cells were transiently transfected with the SOD1 expression constructs (SOD1^WT^ and SOD1^G93A^) fused to EGFP. To verify the SOD1 aggregate formation, we employed three assays: (i) Insolubility in non-denaturing detergents of SOD1 species was assessed by Western blot analysis. After 48 h of transfection, total cell extracts were prepared in 1% Triton buffer in PBS, according to the Western blot protocol described above. The samples were treated with or without reducing agent 100 mM dithiothreitol (DTT) (Thermo Fisher Scientific, R0861). (ii) SOD1 aggregate formation was verified by filter trap assay as detailed in [43]. Briefly, 1 μg/μl protein NSC34 cell extracts treated with or without DTT were filtrated through a 0.2 μm cellulose acetate membrane by using a BRL dot-blot filtration unit. SOD1 aggregates were detected with anti-SOD1 antibody, following the immunological protocol described above. (iii) SOD1 inclusions were monitored by confocal microscopy. In brief, NSC34 cells were seeded on coverslips, transfected with corresponding vectors. After 48 h cells were fixed with 4% paraformaldehyde in PBS for 20 min at room temperature. Fixed cells were imaged with a Leica TCS SP8 confocal microscope with a 40X objective magnification. ImageJ and LAS X were employed to quantify EGFP-positive cells with inclusions and to process the stacked images. (iv) For the isolation of detergent-insoluble protein aggregates, NSC34 cells were seeded in 6-well plates. After 24 h cells were transiently transfected with the SOD1 expression constructs (SOD1^WT^ and SOD1^G93A^) fused to EGFP together with *shCtrl* or shPacer and hPacer-V5. After 48 h of transfection, the cells were collected and centrifuged at 4 °C for 5 min at 900×g the cell pellet was re-suspended with 200 μl buffer TEN (10 mM Tris-HCl, 1 mM EDTA, 10 mM NaCl, pH 8.0) buffer, containing protease inhibitor cocktail 1 X (Thermo Fisher Scientific, A32955) supplemented with 0.5% of non-ionic detergent Nonidet P-40 (NP-40) (EMD Millipore Corp., 9016-45-9), the samples were sonicated 10 s on ice, 40 μl from the total lysate was saved as Input. The remaining lysate was cleared by centrifugation at 800×g for 10 min at 4 °C. To separate the soluble protein fraction and the detergent-insoluble protein aggregates the supernatant was then submitted to centrifugation at 16900×g for 45 min at 4 °C, [44]. The pellet containing the aggregates was washed by addition of TEN buffer and centrifuged again at 16900×g for 45 min at 4 °C. The washed pellet was solubilized in TEN buffer containing 0.5% SDS and protease inhibitor cocktail followed by Western blot analysis under reducing conditions.

### Cell death assay

NSC34 cells stably transduced with *shPacer* and *shCtrl* constructs and then transfected with EGFP, SOD1^WT^, SOD1^G93A^, and hPacer-V5, after 48 h and 72 h the cells were collected and stained with 250 nM of SytoxBLue™ (Thermo Fisher Scientific, S34857). SytoxBlue™ positive cells were monitored by Flow cytometry using a BD FACSVerse™ flow cytometer. FlowJo software version 7.6.1. was used to analyze data.

### Statistical analysis

GraphPad Prism software was used for statistical analysis. All data were analyzed by one-way ANOVA or Student’s t-test. N indicates the number of biological replicates. On all graphs, error bars represent SEM.

## Results

### ALS network analysis links C13orf18/Pacer to autophagy

**Fig. 1:**
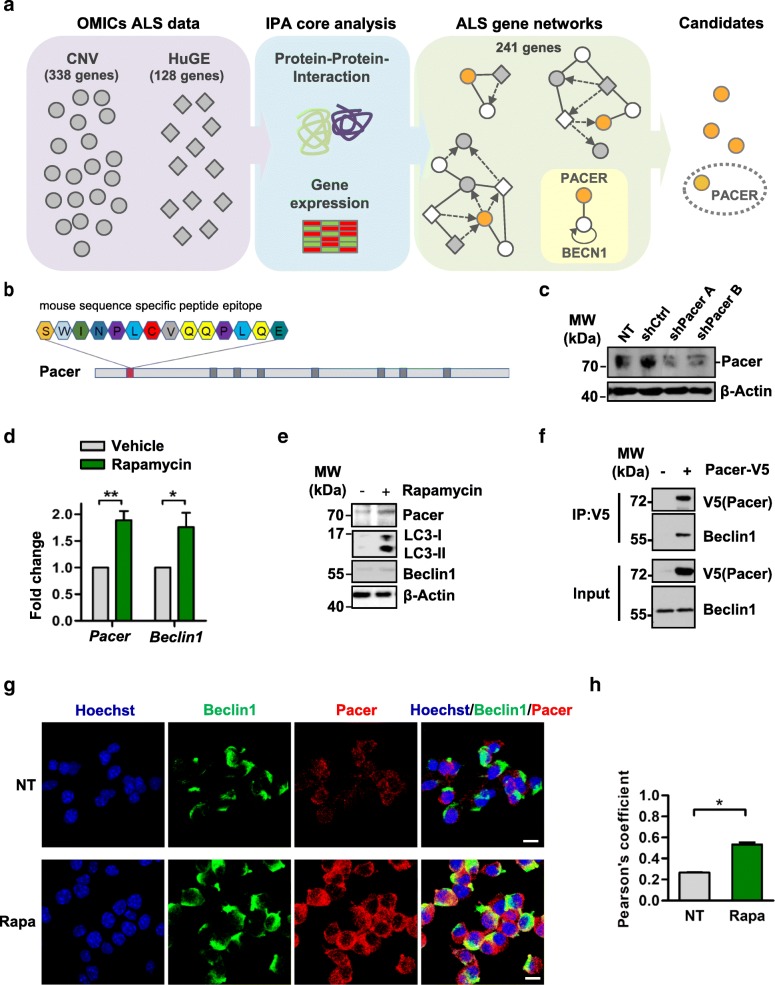
Pacer is identified as a protein involved in autophagy in the context of an ALS disease network. **a** Scheme for the convergent analysis performed for ALS data. CNV, copy number variation; IPA, Ingenuity pathway analysis. **b** Scheme of localization of a total of 8 peptides (dark gray boxes) used to generate an antibody specific for mouse Pacer. Only one peptide (pink box) resulted in the generation of a specific antibody. The aa sequence of this peptide is shown. **c**, The specificity of the antibody generated in **b** was tested by depleting NSC34 cells of Pacer expression using two shRNA constructs targeting mouse *Pacer* mRNA (shPacer A and shPacer B). As a mock control, a scrambled shRNA (shCtrl) construct was used. Pacer was detected by a custom-made antibody by Western blot, a non-transfected (NT) is shown. A representative of 3 independent experiments is shown. **d, e** NSC34 cells were treated with rapamycin for 6 h. **d** mRNA extraction and quantitative PCR was performed (*n* = 4). **e**, Extracts of NSC34 cells were subjected to Western blot analysis. Protein levels of Pacer, Beclin1, and LC3-II were verified. β-Actin serves as a loading control. **f** IP was performed with extracts from HEK293T cells transfected with expression vector for mouse Pacer-V5 or empty control vector for 48 h. IP was performed using the V5 tag. The interaction of V5-tagged Pacer with endogenous Beclin1 was analyzed by Western blot. The inputs and elutions are shown. **g, h** The colocalization of endogenous Pacer and Beclin1 in NSC34 cells under basal (NT) and Rapamycin (Rapa) treatment was was analyzed by **g**, confocal microscopy and **h**, quantified using Pearson’s coefficient (*n* = 3). The nuclei are visualized with Hoechst. Mean and SEM with only statistical significant *p*-values are shown: *, *p* ≤ 0.05

We used a convergent network analysis approach based on [26] to integrate ALS specific high-throughput data, such as HUGE phenopedia and copy number variation (CNV) data (Additional file 1: Table S1) [33–36], with the global protein-protein interaction network. This approach allowed us to build interaction networks with all ALS associated genes, thus uncovering new connections to previously unrecognized genes/proteins as being part of the ALS disease network (Fig. 1a; Additional file 3: Figure S1 and Additional file 2: Table S2). An evidence-based output of 241 genes/proteins organized in 12 ALS-specific subnetworks was obtained (Additional file 3: Figure S1 and Additional file 2: Table S2). We selected Pacer (C13orf18) for further experimental studies, mainly because it was displayed as a component of a subnetwork composed of itself and Beclin1 (Fig. 1a and Additional file 3: Figure S1h), an interaction previously reported in a proteomic study [30]. Beclin1 is a well-characterized core subunit of distinct phosphatidylinositol 3 kinase (PI3K) complexes, which mediate multiple steps during the autophagy process. A role for Beclin1 had been implicated in ALS pathology since its levels were found to be increased in the spinal cord from ALS mouse models, as well as in postmortem sALS spinal cord samples [22, 45, 46]. Through its interaction with Beclin1, Pacer was suggested to functionally connect to the autophagy process [30], which recently was further confirmed by two studies that reported the interaction of Pacer with several components of the Beclin1 complex, including Beclin1, UVRAG, PI3KR4 and PI3KC3 [32, 47]. Hence, we hypothesized that Pacer could play a regulatory role in the autophagy pathway through interaction with Beclin1 and affect ALS pathology through this pathway. Furthermore, an SNP discovered in the 3’UTR of Pacer in a cohort of ALS patients suggests that it could be a modifier of disease [31].

No functional commercial antibody was available to detect mouse Pacer. Hence an antibody targeting endogenous mouse Pacer was custom-made for this study by Abmart. Initially, eight peptides unique to the mouse sequence were tested; however, only peptide 43-SWINPLCVQQPLQE-57 generated an antibody that detected one band at the expected size of 72 kDa in the mouse motoneuron cell line NSC34 by Western blot (Fig. 1b and c). Specificity of the antibody was confirmed by knockdown of mouse Pacer using two shRNA constructs targeting its expression (Fig. 1c and Additional file 4: Figure S2a). The expression level of many autophagy genes, e.g., *Beclin1*, is generally upregulated upon activation of this pathway [48]. We assessed the possible regulation of Pacer expression under autophagy induction in NSC34 cells. Pacer endogenous mRNA and protein levels were increased after treatment with rapamycin, similar to Beclin1 mRNA and protein levels (Fig. 1d and e) as well as LC3B-II protein levels (Fig. 1e). To confirm the interaction of Pacer with Beclin1, we used V5-tagged mouse Pacer (Pacer-V5) in HEK293T cells and performed immunoprecipitation (IP) experiments. Using the V5-tag on Pacer, we observed that endogenous Beclin1 is found in protein complexes with Pacer-V5 (Fig. 1f). By using the custom-made Pacer antibody, we confirmed endogenous Pacer/Beclin1 colocalization in NSC34 cells (Fig. 1g). The Pearson’s correlation coefficient between the two proteins was found to increase from 0.26 under basal conditions to 0.53 under autophagy activation with rapamycin (Fig. 1h).

To investigate the presence of *Pacer* mRNA in tissues relevant to ALS pathogenesis, we assessed *Pacer, Rubicon* and *Beclin1* mRNA levels by quantitative PCR in different mouse tissues (cortex, cerebellum, hippocampus, spinal cord, muscle, and liver) obtained from 100 days-old wild-type C57BL/6 mice (4 males and 4 females). The three genes displayed elevated mRNA expression in the central nervous system (CNS) when compared to muscle or liver (Additional file 4: Figure S2b). *Pacer* and *Rubicon* showed higher expression in the spinal cord and cortex, while *Beclin1* expression was elevated in the hippocampus, confirming previous findings (Additional file 4: Figure S2b) [49].

The same antibody described above was subsequently used to detect endogenous mouse Pacer in mouse spinal cord tissue. In the lumbar spinal cord of 60 days old wild-type mice, Pacer cell-type expression was investigated by immunofluorescent staining and confocal microscopy. Strikingly, Pacer appeared to be expressed mainly in neurons in the spinal cord, with no obvious detection in astrocytes (Additional file 4: Figure S2c).

### Pacer levels are decreased in ALS pathology

**Fig. 2:**
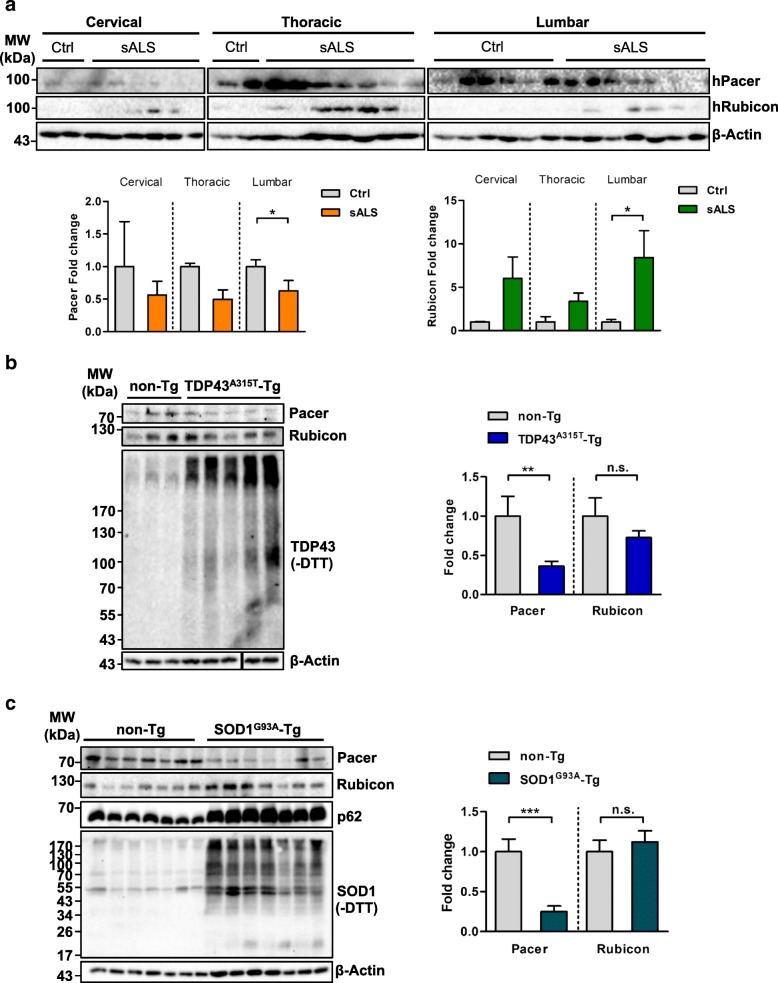
Pacer levels are reduced during ALS pathology. **a** Human Pacer (hPacer) and human Rubicon (hRubicon) protein levels were determined in post-mortem spinal cord sections from sALS patients and age-matched control subjects. Left panel, cervical spinal cord section with Controls *n* = 2 and sALS patients *n* = 6; middle panel, thoracic spinal cord section with Controls *n* = 2 and sALS patients *n* = 7; and right panel, lumbar spinal cord section with Controls *n* = 6 and sALS patients *n* = 7. β-Actin serves as a loading control. Densitometric quantifications of hPacer and hRubicon normalized to β-Actin levels are shown. **b** Pacer and Rubicon protein levels were determined in lumbar spinal cord samples of late symptomatic TDP43^A315T^ transgenic mice (TDP43^A315T^-Tg, *n* = 5) and their non-transgenic littermate controls (*n* = 3), respectively. TDP43 aggregate levels under non-reducing (−DTT) conditions are shown as positive controls. β-Actin serves as a loading control. Densitometric quantifications of Pacer protein levels normalized to β-Actin levels are shown. **c**, Pacer and Rubicon protein levels were determined in the lumbar spinal cord of late symptomatic SOD1^G93A^ transgenic mice (SOD1^G93A^-Tg) and their non-transgenic littermate controls (both groups *n* = 7). p62 protein levels were detected as a positive control of impaired autophagy. SOD1 aggregate levels under non-reduced (−DTT) conditions are shown as a positive control for SOD1^G93A^-Tg mice. β-Actin serves as a loading control. Densitometric quantifications of Pacer protein levels normalized to β-Actin levels are shown. In **a**-**c** Statistical analyses were performed using Student’s t-test. Mean and SEM with only statistical significant p-values are shown: *, *p* ≤ 0.05; **, *p* ≤ 0.01; and ***, *p* ≤ 0.001

We next evaluated possible alterations in Pacer levels in human postmortem spinal cord samples derived from patients with sALS compared to non-ALS patients (Additional file 5: Table S3). We found a significant decrease in Pacer protein levels in the lumbar spinal cord of sALS patients compared to age-matched healthy control subjects (Fig. 2a). A similar trend was observed in the thoracic spinal cord, yet no tendency was found in the cervical spinal cord (Fig. 2a). However, for both cervical and thoracic sections tissues from only two control cases were available. Interestingly, in sALS patients the decrease of Pacer protein levels correlated with the increase of Rubicon protein levels in the same patient, a phenomenon observed in all spinal cord sections (Fig. 2a). While *Pacer* mRNA levels were not altered, Rubicon mRNA levels were increased in the lumbar spinal cord of sALS patients (Additional file 6: Figure S3a and b). These findings indicate that at least the lumbar region of the spinal cord of sALS patients displays a significant loss of Pacer protein levels, suggesting a possible involvement of Pacer in ALS pathogenesis.

We then evaluated Pacer levels in tissue derived from two ALS mouse models, TDP43^A315T^ and SOD1^G93A^ transgenic mice (TDP43^A315T^-Tg and SOD1^G93A^-Tg, respectively) [37, 38, 50]. We assessed Pacer mRNA and protein levels in the lumbar spinal cords of late symptomatic transgenic mice compared to their respective non-transgenic (non-Tg) littermates in the age range of 159 to 189 days for TDP43^A315T^-Tg mice and 131 to 152 days for SOD1^G93A^-Tg mice. Pacer protein levels were significantly diminished in the spinal cords of symptomatic TDP43^A315T^-Tg and SOD1^G93A^-Tg mice compared to their respective non-Tg controls (Fig. 2b and c). In comparison, Rubicon protein levels were not affected in either mouse model (Fig. 2b and c). Both *Pacer* and *Rubicon* mRNA levels were not altered in either mouse model (Additional file 6: Figure S3c and S3d).

**Fig. 3:**
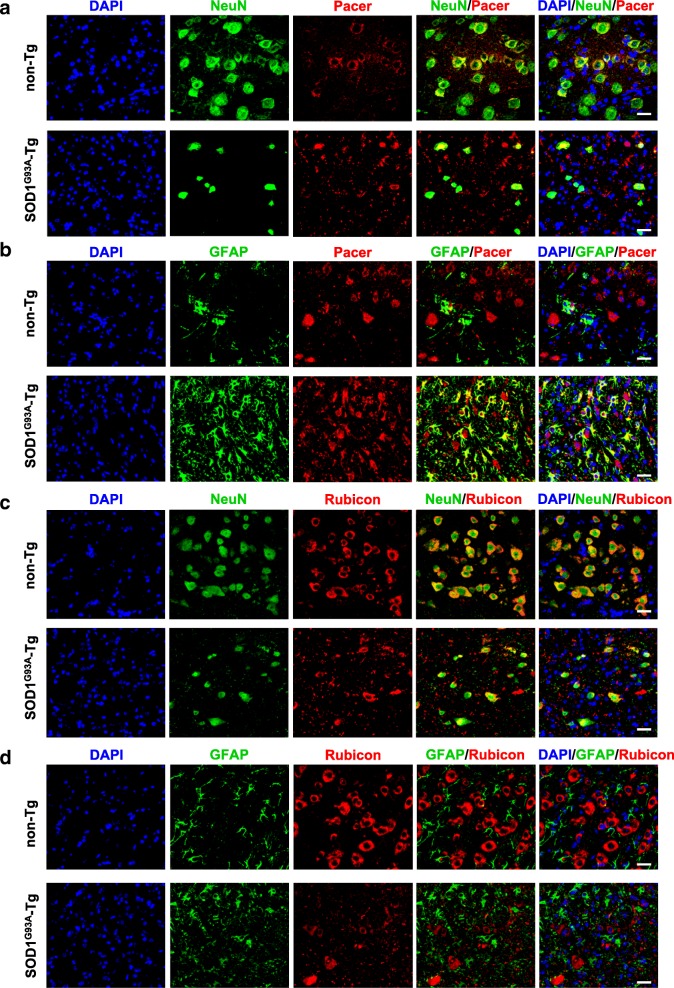
Pacer and Rubicon localization in the spinal cord of symptomatic SOD1^G93A^ transgenic mice. Confocal microscopy of lumbar spinal cord sections of late symptomatic (138 to 156 days old) SOD1^G93A^ transgenic mice (SOD1^G93A^-Tg) compared to age-matched non-transgenic controls (non-Tg). Z-stack of confocal images, detection of Pacer, the neuronal marker NeuN in **a**, or the astrocytic marker GFAP in **b**, detection of Rubicon, the neuronal marker NeuN in **c**, or the astrocytic marker GFAP in **d**. **a-d,** Nuclei are stained with DAPI. Scale bar: 20 μm

To investigate if the localization of Pacer to neurons is affected under disease condition, we performed immunofluorescence staining and confocal microscopy of lumbar spinal cord sections of symptomatic SOD1^G93A^-Tg mice and their age-matched littermate controls (in the age range from 138 to 156 days). We focused on the ventral horn region of the spinal cord where motoneurons are found. In non-Tg animals, Pacer localized mainly to neurons, including motoneurons in the ventral horn (Fig. 3a and b, upper panels). In symptomatic SOD1^G93A^-Tg mice less overall Pacer staining in neurons was found, possibly due to a lower number of surviving neurons in the ventral horn of the spinal cord (Fig. 3a and b, lower panels). Surprisingly, in SOD1^G93A^-Tg mice Pacer also localized to astrocytes, which was not observed in non-Tg control mice (Fig. 3b, lower panel). Like Pacer, Rubicon was also preferentially expressed in neurons, in non-Tg control mice (Fig. 3c and d, upper panels). Similar to Pacer, Rubicon staining was diminished in neurons in symptomatic SOD1^G93A^-Tg mice possibly due to a motoneuronal loss in the ventral horn of the spinal cord (Fig. 3c and d, lower panels).

To assess if the decrease in Pacer levels under disease conditions is simply due to the loss of motoneurons in the symptomatic spinal cord or if Pacer levels are affected already earlier, we evaluated Pacer levels also in pre-symptomatic SOD1^G93A^-Tg and age-matched non-Tg mice (60 days). Pacer levels were reduced in pre-symptomatic SOD1^G93A^-Tg, similar to Rubicon, Beclin1 and LC3-II (Additional file 7: Figure S4a), which could be due to the activation of the autophagy pathway. Additionally, the localization of Pacer in the spinal cord of pre-symptomatic SOD1^G93A^-Tg and age-matched non-Tg mice (60 days) was investigated. Pacer was found to localize mainly to neurons in both Tg and non-Tg animals (Additional file 7: Figure S4b and S4c). No staining was observed in astrocytes in pre-symptomatic mice (Additional file 7: Figure S4c). Similar as reported in [21, 51], we used metalloproteinase-9 (MMP9) as a marker for vulnerable neurons known to degenerate in ALS mouse models. Pacer was found to localize to MMP9-positive neurons in both SOD1^G93A^-Tg and non-Tg mice (Additional File 8: Fig. S5a and S5b), nevertheless, in SOD1^G93A^-Tg mice, the immunofluorescence labeling of Pacer appeared to be decreased compared to non-Tg mice (Additional file 8: Figure S5b). Together with our results in Western blot, these data suggest that vulnerable neurons display a decrease in Pacer expression already at presymptomatic stages in the SOD1^G93A^ ALS mouse model.

### Diminished Pacer levels impair autophagy

**Fig. 4:**
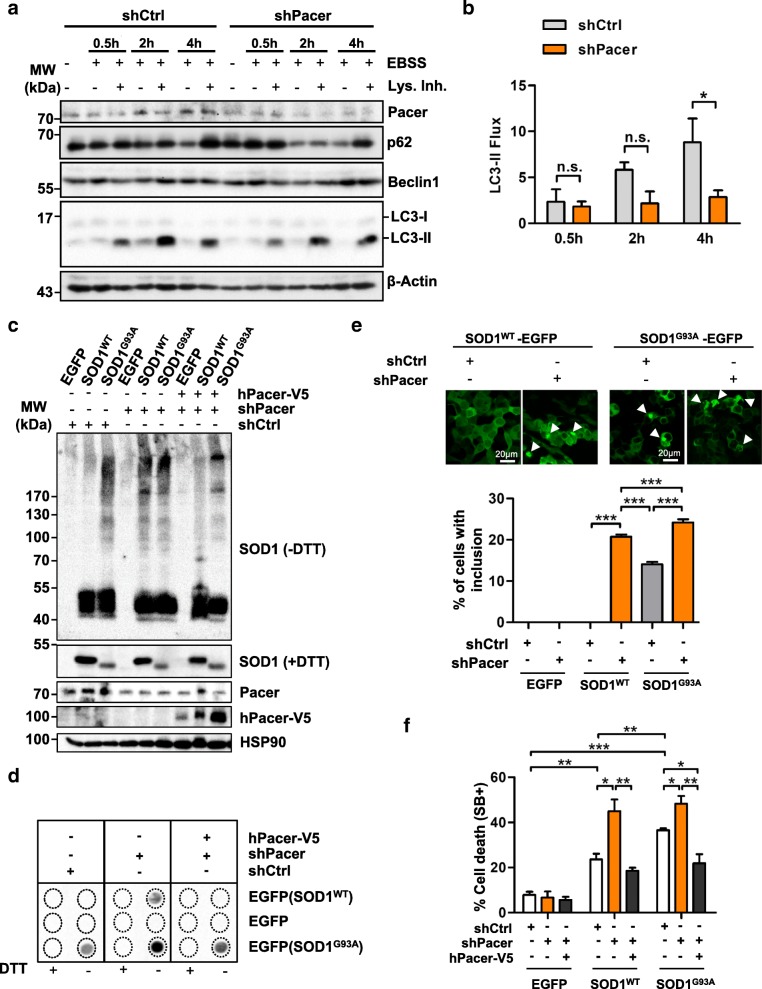
Depletion of Pacer impairs autophagosome formation and promotes SOD1 aggregation. **a** Autophagy flux under *Pacer* knockdown. Cells were treated with EBSS medium or/and lysosome inhibitors (Lys. Inh.) for 0.5, 2 and 4 h. Cell extracts were subjected to Western blot. As a mock control, a scrambled shRNA (shCtrl) construct was used. Pacer, Beclin1, p62 and LC3-II formation levels were determined. β-Actin serves as a loading control**. b** Densitometric quantifications of LC3-II flux (n = 3). One-way ANOVA and Bonferroni’s post hoc tests were performed.Mean and SEM with only statistically significant p-values are shown: *, *p* ≤ 0.05. **c-f**, NSC34 cells depleted of Pacer were transiently co-transfected with expression vectors for human wild-type or mutant SOD1^G93A^ fused to EGFP. When indicated, human Pacer (hPacer-V5) was co-expressed. **c** and **d**, after 48 h, SOD1 aggregation was assessed under non-reducing (−DTT) conditions. Cell extracts were prepared in 1% Triton X-100 buffer or 1% SDS buffer for Western blot and filter trap assays, respectively. In **c** HSP90 serves as a loading control. **e** SOD1 inclusions in NSC34 cells were assessed by confocal microscopy. Percentages of cells with SOD1^WT^-EGFP or SOD1^G93A^-EGFP inclusions are shown. **f** Percentage of cell death was quantified at 72 h (SytoxBlue positive, SB+) in NSC34 stable lines expressing shPacer or shCtrl transiently transfected with EGFP, SOD1^WT^or SOD1^G93A^, and hPacer-V5. In **e** and **f** statistical analyses were performed using one-way ANOVA and Bonferroni’s post-hoc tests. Mean and SEM with only statistically significant p-values are shown: *, *p* ≤ 0.05; **, *p* ≤ 0.01; and ***, *p* ≤ 0.001

Since Pacer was indicated by our network analysis to associate with Beclin1 and its levels were decreased in ALS-affected tissue, we assessed the possible role of Pacer expression in autophagy using a loss-of-function approach. To determine if the loss of Pacer affects the autophagy process, we monitored autophagy flux in NSC34 cells deficient for Pacer. We depleted Pacer in NSC34 cells using short-hairpin RNAs (*shPacer* A and B) (Fig. 1c and Additional file 4: Figure S2a) and determined the levels of key autophagy markers in NSC34 cells under serum deprivation conditions (EBSS) in the presence or absence of lysosomal inhibitors. Depletion of Pacer decreased LC3-II flux in NSC34 cells (Fig. 4a and b), suggesting an impairment of the autophagy process. Autophagy substrate p62 and Beclin1 were not significantly affected (Fig. 4a and Additional file 9: Figure S6a and S6b). Taken together, our results suggest that reduced levels of Pacer negatively affect the autophagy pathway.

### Pacer repression promotes SOD1 aggregate accumulation and triggers cell death

To determine the consequences of reduced Pacer levels in the ALS pathogenesis which we observed in vivo (Fig. 2), we assessed the impact of its repression on mutant SOD1 aggregation and neuronal cell death. Knocking down Pacer in NSC34 cells led to increased mutant SOD1 disulfide-dependent aggregation (Fig. 4c). Interestingly, we also observed the spontaneous aggregation of wild-type SOD1 upon loss of Pacer (Fig. 4c), a phenomenon described in sALS cases [52–54]. Similar results were obtained by determining the levels of Nonidet P-40 (NP-40) detergent-insoluble protein aggregates (Additional file 9: Figure S6c), as well as by filter trap, an assay that detects protein aggregates by size using a cellulose acetate filter membrane with 200 nm pores (Fig. 4d). Since our shRNA constructs target mouse Pacer exclusively, we restored Pacer levels by exogenous expression of human Pacer (hPacer-V5) (Additional file 9: Figure S6d and S6e). The re-expression of hPacer-V5 restored SOD1 aggregation levels to a similar extent to those observed in control cells (Fig. 4c, d and Additional file 9: Figure S6c). Furthermore, confocal microscopy of SOD1 confirmed these findings. Depletion of Pacer in NSC34 cells led to an increased number of inclusions formed by SOD1^G93A^ (Fig. 4e and Additional file 10: Figure S7), whereas it had dramatic effects on SOD1^WT^, which built large de novo inclusions similar in size to aggregates observed for mutant SOD1 (Fig. 4e and Additional file 10: Fig. S7). No effect of Pacer depletion on EGFP alone was observed (Fig. 4e and Additional file 10: Figure S7). Since impaired autophagy and increased protein aggregation are correlated with neuronal death, we investigated the effect of knocking down *Pacer* in NSC34 cells expressing wild-type or mutant SOD1. We found that the depletion of Pacer sensitizes cells to SOD1^WT^ or SOD1^G93A^ toxicity, whereas cells expressing EGFP alone are unaffected (Fig. 4f and Additional file 10: Figure S7a and S7b). In comparison, the depletion of Rubicon did not result in cell death in NSC34 cells expressing mutant SOD1 (Additional file 10: Figure S7c and S7d). Furthermore, the reconstitution with hPacer-V5 rescued the survival of cells expressing SOD1^WT^ or SOD1^G93A^ (Fig. 4f). Human Pacer expression even improved the survival rate when compared to cells expressing scrambled shRNA construct (*shCtrl* cells) in the presence of SOD1^G93A^ (Fig. 4f). Taken together these results indicate an important role for Pacer in maintaining proteostasis in motoneurons by promoting SOD1 aggregate removal and sustaining motoneuron survival.

## Discussion

Neurodegenerative diseases, such as AD, PD, and ALS, are multifactorial, involving a combination of genetic and environmental factors, as well as age as the primary risk factor. Genetic studies in ALS have made significant advances in the understanding of disease pathogenesis by using whole genome or whole exome sequencing strategies (reviewed in [2]). However, the primary cause of approximately half of fALS cases and the majority of sALS cases remains unexplained. The use of systems biology approaches to study neurodevelopmental and neurodegenerative diseases has recently proven to aid our understanding of underlying disease mechanisms by unraveling new genes, pathways or subnetworks responsible for an illness which would not have been recognized using traditional approaches. Here we describe Pacer, a protein previously proposed as a tumor suppressor and cancer biomarker [55–57] and recently reported to be associated with autophagy [32], as being immersed in the ALS disease network. We performed a convergent analysis by merging all ALS data available to uncover pathways and associated new genes involved in the disease. By retrieving and integrating data from different model systems and patient studies, experimental bias was filtered out, and only relevant mechanisms may be uncovered. From the list of genes obtained through this analysis, we selected the protein Pacer, previously known as C13orf18, based on its putative role in autophagy, a compromised pathway during ALS pathogenesis. At the time, the only data available for Pacer was based on a proteomic study of the autophagy network, which suggested a role in the autophagy pathway via interaction with Beclin1 [30]. Furthermore, a connection to ALS was suggested by the identification of an SNP in the 3’UTR of the *Pacer* gene in a cohort of sALS patients [31]. The specific consequence of the SNP in the 3’UTR of the Pacer gene in ALS pathogenesis, however, need to be more explored in future studies. The likely presence of variants in the UTR or intronic regions of ALS genes has been exemplified by the most common genetic modification known to cause ALS, C9orf72 intronic repetitions [58]. Furthermore, variants in untranslated regions of well-known ALS-causing genes, including *SOD1*, *TARDBP*, *FUS* and *UBQLN2*, have been recently reported [59]. Although these variants do affect the amino acid sequence of the affected the protein, they can have important effects on other aspects, such as expression level. For instance, Al-Chalabi and co-workers showed that variants in the 3’UTR of the *FUS* gene found in Italian ALS patients promote mislocalization of the FUS protein and result in a dramatically increased expression of this protein [60, 61]. Similarly, variants found in the 3’UTR of the *TARDBP* gene also regulate its expression by affecting the RNA stability of its transcripts [62]. Here, we found Pacer levels to be decreased in sALS patients, similarly to two fALS mouse models, hence Pacer may participate in a transversal mechanism to protect neurons against disease.

Recently, Pacer was described as a positive regulator of autophagosome maturation, through its binding to the functionally distinct UVRAG-Beclin1-Vsp34 and UVRAG-HOPS complexes [32]. Pacer is proposed to target both protein complexes to the autophagosome membrane for activation [32]. Beclin1 is a core subunit of distinct PI3K complexes, which mediate multiple steps during the autophagy process. Beclin1 levels were found to be increased in the spinal cord from ALS mouse models, as well as in postmortem sALS spinal cord samples [22, 45, 46]. We have shown that haploinsufficiency of Beclin1 in a mutant SOD1 mouse model increases the lifespan of double-transgenic mice, despite the increase in SOD1 aggregates [22], consistent with recent findings in Atg7 deficient animals [21]. In another study, however, targeting Beclin1 in two different mouse models of ALS showed opposite results [46]. Here, we show that Pacer is expressed in the CNS, particularly in neurons in the spinal cord, suggesting it to have an essential role in neuronal cells in this tissue. We show that Pacer levels are decreased in the lumbar region of the spinal cord in two fALS mouse models in the late stage of the disease and biopsies of sALS patients. Furthermore, we found that while Pacer protein levels decrease in the late stage of disease in both mice and humans, Rubicon protein levels are maintained in mice or increased in humans. In histological studies, we found that in late symptomatic SOD1^G93A^ transgenic mice the Pacer staining was diminished in motoneurons, probably due to the loss of motoneurons, whereas its expression was augmented in astrocytes. Progressive reactive astrogliosis is a feature of the ALS pathogenesis, mainly found surrounding degenerating neurons [63]. Changes in the expression of autophagy proteins specifically in astrocytes during ALS have not been reported to our knowledge. Our finding that Pacer is also expressed in astrocytes in the symptomatic stage of an ALS mouse model whereas it is only found in neurons in the presymptomatic stage could imply a role of Pacer in the inflammatory response of the astrocytes during disease progression, however further investigastions are necessaries to clarify this point.

Similar to Pacer, Rubicon also was localized primarily to neurons in the lumbar spinal cord in non-Tg mice. Nevertheless, in late symptomatic SOD1^G93A^-Tg animals Rubicon localization to motoneurons was diminished similarly to Pacer. However, its expression was augmented in another cell type, other than astrocytes, possibly microglia, which remains to be investigated further. Hence, our results suggest that the expression of both Pacer and Rubicon is highly dynamic and can change depending on the cell type as well as under ALS disease condition. Additionally, our results raise caution for the interpretation of Western blot results which represent overall protein levels and cannot distinguish changes of expression in various cell types, such we have observed in our histological analysis for both Pacer and Rubicon.

To investigate whether the loss of Pacer during the late stage of disease signifies a possible involvement in ALS pathogenesis or is only representative of the loss of vulnerable neurons during the course of the disease, we also determined its levels and localization during the presymptomatic stage of the disease at age 60 days in the SOD1^G93A^ mouse model. We found that total Pacer levels are already significantly decreased in presymptomatic SOD1^G93A^-Tg mice, while we do not observe any neuronal loss or signs of astrogliosis, as has been reported [64]. In the presymptomatic stage, Pacer appears to be expressed exclusively in neurons in the lumbar spinal cord in SOD1^G93A^-Tg mice, comparable to non-Tg littermate controls, nevertheless its overall signal seemed reduced in transgenic mice, possibly reflecting its decreased total protein level observed in Western blot. To show that Pacer localizes to the neurons that are lost later during disease progression, we performed co-staining of Pacer with MMP9, a marker for vulnerable motoneurons [21, 51]. Indeed, we found that Pacer colocalizes with MMP9, hence our data suggests a correlation between the loss of function of Pacer in spinal cord motoneurons and ALS pathogenesis.

To study the role of Pacer during ALS pathogenesis, we used the mouse motoneuron-like cell line NSC34. We demonstrated that *Pacer* expression is up-regulated on the transcriptional and translational level upon autophagy induction, resembling the behavior of other autophagy genes [65, 66]. Furthermore, we confirmed the interaction between Pacer and Beclin1. Comparable results were obtained by Cheng et al. in U2OS cells recently [32]. Pacer shares highest sequence homology with Rubicon, which has become, in recent years, known as an important regulatory protein of autophagy and endocytosis, as well as of LC3-associated phagocytosis [67–71]. Pacer and Rubicon are part of the same protein family due to their shared Rubicon homology (RH) domain. Interestingly, they were reported to perform opposing functions in the autophagy pathway, Rubicon as a negative regulator of the UVRAG-Beclin1-Vsp34 and UVRAG-HOPS complexes through its binding to UVRAG, while Pacer antagonizes Rubicon by competing for UVRAG binding, hence positively influencing autophagy activity [32]. In concordance with these results obtained in HEK293T cells [32], we also find that motoneurons depleted of Pacer are impaired in their autophagy flux.

In the current literature, the dependence of motoneurons and the neuromuscular junction (NMJ) on autophagic activity is actively discussed [21, 72, 73]. On one hand due to the importance of autophagy in maintaining the high metabolic rate of motoneurons and on the other hand the requirement of motoneuron autophagy in preserving neuromuscular innervation [21]. Motoneurons have been shown to be extremely dependent on an efficient autophagy flux, referring to an accurate degradation of the formed autophagosomes in the lysosome [21, 72]. Furthermore, during aging a progressive impairment of late endolysosomal processes is observed in neurons, resulting in increased cellular stress owing to the continuous input of new autophagosomes to cope with the turnover of organelles and other cargos (reviewed in [74, 75]). In ALS a progressive decrease in autophagic/endocytic activity in affected motoneurons has been described in fALS mouse models since early stages of the disease [76, 77]. In sALS patients, the levels of autophagy markers in spinal cord postmortem samples were described to be increased, including levels of Beclin1, LC3-II and the autophagy-substrates p62, protein aggregates, and unfunctional organelles, correlating with a dysregulation of the pathway [45, 46]. Finally, mutations in several autophagy-related genes were found associated with fALS, further supporting the notion that autophagy dysfunction is a significant pathogenic mechanism of disease (reviewed in [8]). In our study, we found that Pacer loss of function in a motoneuron cell line results in an augmentation of mutant SOD1 aggregation, as well as an unexpectedly drastic de novo aggregation of wild-type SOD1. This phenomenon may be explained by the disruption of the autophagy process due to loss of Pacer since both wild-type and mutant SOD1 are autophagy substrates [78]. Together these results suggest that Pacer is required for protein aggregate degradation under physiologic and disease conditions and that its loss could lead to a disruption of proteostasis maintenance. In this context, we found that depleting cells of Pacer not only caused the accumulation of protein aggregates in our cellular model but also sensitized cells to death induced by SOD1^WT^ and SOD1^G93A^ expression. Furthermore, the re-expression of Pacer protects against this sensitization and even improved the survival of the NSC34 cells expressing SOD1^G93A^ protein. Overall, these results suggest that Pacer could be an important regulator of the cellular proteostasis and neuronal survival including of vulnerable motoneurons.

## Conclusions

In summary, our results suggest a role for Pacer as an essential component of the autophagy machinery in neurons, especially motoneurons, where it promotes the clearance of protein aggregates through the autophagy pathway thereby maintaining proteostasis and sustaining neural survival. Hence, our results suggest Pacer as a potential new candidate to study its therapeutic effect in ALS. Furthermore, our experimental results also validate our unbiased bioinformatic approach, which initially suggested Pacer as a protein involved in ALS pathology through a connection with the autophagy pathway. Like Pacer, many previously uncharacterized genes were identified as part of a functional context associated with complex disorders by these type of systems biology analyses [29, 79, 80]. This approach promises to accelerate gene and/or pathway discovery in complex diseases and can help guide experimental studies that may lead to the identification of biomarkers or therapeutic targets.

## Additional files

Additional file 1: **Table S1.** Genes related to ALS in CNV and HUGE databases. (DOCX 31 kb)

Additional file 2: **Table S2.** Genes in 12 ALS associated networks. (DOCX 29 kb)

Additional file 3: **Figure S1.** ALS convergent analysis subnetworks. a-l, ALS disease subnetworks generated by IPA as an output for the convergent analysis approach presented in Fig. 1a. (PPTX 603 kb)

Additional file 4: **Figure S2.** Pacer is expressed in neurons in the spinal cord of wild-type mice **a**, mRNA levels of Pacer in NSC34 cells depleted of Pacer using shRNA constructs (shRNA A and shRNA B) compared to a scrambled control (shCtrl) were determined by real-time. 18s rRNA levels were used for normalization. Statistical analyses were performed using Student’s t-test. Mean, and SEM with only statistically significant p-values are shown: ***, p ≤ 0.001. **b**, mRNA levels of Pacer, Rubicon and Beclin1 were determined by quantitative PCR in the spinal cord, cortex, hippocampus, cerebellum, muscle, and liver of wild-type C57BL/6 mice (n=8, 4 females, 4 males). mRNA levels in the liver are used as a reference. c, Confocal microscopy of lumbar spinal cord sections of wild-type mice. Z-stack of confocal images, detection of Pacer, the neuron marker NeuN, or the astrocytic marker GFAP, and DAPI detection by immunofluorescence in C57BL/6 46 mice. Scale bars: 300 μm, and 20 μm. Doted inset indicates where higher magnification images were taken. (PPTX 971 kb)

Additional file 5: **Table S3.** Clinical and histopathological data of control and sporadic ALS cases. (DOCX 58 kb)

Additional file 6: **Figure S3.** *Pacer* mRNA levels in the lumbar spinal cord from sALS patients and fALS mouse models. **a**, Human Pacer (hPacer) and **b**, human Rubicon (hRubicon) mRNA expression was determined by qPCR in postmortem spinal cord sections from sALS patients and age-matched control subjects. Left panel, cervical spinal cord section with Controls n=2 and sALS patients n=6; middle panel, thoracic spinal cord section with Controls n=2 and sALS patients n=7; and right panel, lumbar spinal cord section with Controls n=6 and sALS patients n=7. β-Actin mRNA levels were used for normalization. **c**, Pacer and Rubicon mRNA expression was determined by qPCR in lumbar spinal cord samples of late symptomatic TDP43A315T transgenic mice (TDP43A315T-Tg, n=5) and their non-transgenic littermate controls (n=3), respectively. β-Actin levels were used for normalization. **d**, Pacer and Rubicon mRNA expression was determined in the lumbar spinal cord of late symptomatic SOD1G93A transgenic mice (SOD1G93A-Tg) and their non-transgenic littermate controls (both groups, n=7). 18S RNA levels were used for normalization. (PPTX 362 kb)

Additional file 7: **Figure S4.** Pacer levels and localization in the spinal cord of presymptomatic SOD1^G93A^ transgenic mice. **a**, Pacer, Rubicon, Beclin1, p62, LC3II protein levels were determined in the lumbar spinal cord of presymptomatic 47 (60 days old) SOD1G93A transgenic mice (SOD1G93A-Tg, n=4) and their non-transgenic littermate controls (n=5). SOD1 human levels are shown as a positive control for SOD1G93A-Tg mice. β-Actin serves as a loading control. Densitometric quantifications of Pacer, Rubicon, Beclin1, p62 and LC3II protein levels normalized to β-Actin levels are shown. **b**, Confocal microscopy of lumbar spinal cord sections of presymptomatic (60 days old) SOD1G93A transgenic mice (SOD1G93A-Tg, lower panel) compared to age-matched non-transgenic controls (non-Tg, upper panel). Z-stack of confocal images, detection of Pacer, the neuronal marker NeuN in **b**, or the astrocytic marker GFAP in **c**. **b** and **c**, Nuclei are stained with Hoechst. Scale bar: 30 μm. (PPTX 2790 kb)

Additional file 8: **Figure S5.** Pacer is expressed in MMP9-positive cells in the presymptomatic spinal cord of SOD1^G93A^ transgenic mice. **a**, Z-stack confocal images of Pacer with MMP9 in lumbar spinal cord sections of non-transgenic controls (non-Tg, 60 days old) and **b**, presymptomatic (60 days old) SOD1G93A transgenic mice (SOD1G93A-Tg) at 10X (upper panel, scale bar: 300 μm), 40X (middle panel, scale bar: 30 μm) and 63X (lower panel, scale bar: 15 μm) magnification. Doted insets indicate where higher magnification images were taken. (PPTX 2260 kb)

Additional file 9: **Figure S6.** Pacer depletion results in detergent insoluble SOD1 aggregate accumulation. **a**-**b**, Densiometric quantification of p62 and Beclin1 levels in the autophagic flux as shown in Fig. 4a. NSC34 cells depleted of Pacer and a scrambled shRNA control (shCtrl) construct were compared (n=3). â-Actin 48 served as a loading control. Statistical analyses were performed using one-way ANOVA and Bonferroni’s post-hoc tests. Mean and SEM are shown. **c**, NSC34 cells depleted of Pacer were transiently co-transfected with expression vectors for human wild-type or mutant SOD1G93A fused to EGFP. When indicated, human Pacer (hPacer-V5) was co-expressed. NP40-detergent insoluble protein aggregates (NP40 insoluble) were prepared as described in materials and methods. The input is shown as a reference. â-Actin levels in the input serve as a loading control. **d**-**e**, Alignment of **d** shRNA A and **e** shRNA B to the corresponding target region in mRNA of mouse Pacer and human Pacer. 100% identity between shRNAs A and B to their respective mouse Pacer target sequences. No significant similarity is found to the corresponding region of human Pacer mRNA. (PPTX 2110 kb)

Additional file 10: **Figure S7.** Depletion of Pacer leads to SOD1 aggregate accumulation. **a**, NSC34 cells were transiently transfected with constructs for shCtrl, shPacer, EGFP, SOD1WT-EGFP and SOD1G93A-EGFP, inclusions are shown with white arrows (Representative images of 3 independents experiments). Scale bar 40 μM. **b**-**c**, Stable NSC34 cell lines expressing **b** shPacer and **c** shRubicon were established. Knockdown was confirmed by Western blot. HSP90 and b-Actin were used as loading controls, respectively. **d**, Percentage of cell death at 48 h (SytoxBlue positive, SB+) in NSC34 stable lines expressing shPacer, shRubicon, and shCtrl constructs. Cells were transiently transfected with plasmids for EGFP or SOD1G93A-EGFP. In d statistical analyses were performed using one- 49 way ANOVA and Bonferroni’s post-hoc tests. Mean and SEM with only statistically significant p-values are shown: *, p ≤ 0.05; and **, p ≤ 0.01. (PPTX 777 kb)

## Abbreviations

ALS: Amyotrophic lateral sclerosis
FTD: Frontotemporal dementia
SOD1: Superoxide dismutase 1
TDP43: TAR DNA-binding protein 43

## References

[1] Brown RH, Al-Chalabi A (2017). Amyotrophic lateral sclerosis. N Engl J Med.

[2] Al-Chalabi A, van den Berg LH, Veldink J. Gene discovery in amyotrophic lateral sclerosis: implications for clinical management. Nat Rev Neurol. 2016. 10.1038/nrneurol.2016.182 27982040

[3] Taylor JP, Brown RH, Cleveland DW (2016). Decoding ALS: from genes to mechanism. Nature.

[4] Sreedharan J, Blair IP, Tripathi VB, Hu X, Vance C, Rogelj B, Ackerley S, Durnall JC, Williams KL, Buratti E (2008). TDP-43 mutations in familial and sporadic amyotrophic lateral sclerosis. Science.

[5] Renton AE, Majounie E, Waite A, Simon-Sanchez J, Rollinson S, Gibbs JR, Schymick JC, Laaksovirta H, van Swieten JC, Myllykangas L (2011). A hexanucleotide repeat expansion in C9ORF72 is the cause of chromosome 9p21-linked ALS-FTD. Neuron.

[6] Majounie E, Renton AE, Mok K, Dopper EG, Waite A, Rollinson S, Chio A, Restagno G, Nicolaou N, Simon-Sanchez J (2012). Frequency of the C9orf72 hexanucleotide repeat expansion in patients with amyotrophic lateral sclerosis and frontotemporal dementia: a cross-sectional study. Lancet Neurol.

[7] Medinas DB, Valenzuela V, Hetz C (2017). Proteostasis disturbance in amyotrophic lateral sclerosis. Hum Mol Genet.

[8] Gao FB, Almeida S, Lopez-Gonzalez R (2017). Dysregulated molecular pathways in amyotrophic lateral sclerosis-frontotemporal dementia spectrum disorder. EMBO J.

[9] Yang Z, Klionsky DJ (2010). Eaten alive: a history of macroautophagy. Nat Cell Biol.

[10] Galluzzi L, Baehrecke EH, Ballabio A, Boya P, Bravo-San Pedro JM, Cecconi F, Choi AM, Chu CT, Codogno P, Colombo MI (2017). Molecular definitions of autophagy and related processes. EMBO J.

[11] Kourtis N, Tavernarakis N (2009). Autophagy and cell death in model organisms. Cell Death Differ.

[12] Hara T, Nakamura K, Matsui M, Yamamoto A, Nakahara Y, Suzuki-Migishima R, Yokoyama M, Mishima K, Saito I, Okano H (2006). Suppression of basal autophagy in neural cells causes neurodegenerative disease in mice. Nature.

[13] Komatsu M, Ueno T, Waguri S, Uchiyama Y, Kominami E, Tanaka K (2007). Constitutive autophagy: vital role in clearance of unfavorable proteins in neurons. Cell Death Differ.

[14] Komatsu M, Waguri S, Chiba T, Murata S, Iwata J, Tanida I, Ueno T, Koike M, Uchiyama Y, Kominami E (2006). Loss of autophagy in the central nervous system causes neurodegeneration in mice. Nature.

[15] Vidal RL, Matus S, Bargsted L, Hetz C (2014). Targeting autophagy in neurodegenerative diseases. Trends Pharmacol Sci.

[16] Menzies FM, Fleming A, Caricasole A, Bento CF, Andrews SP, Ashkenazi A, Fullgrabe J, Jackson A, Jimenez Sanchez M, Karabiyik C (2017). Autophagy and neurodegeneration: pathogenic mechanisms and therapeutic opportunities. Neuron.

[17] Maruyama H, Morino H, Ito H, Izumi Y, Kato H, Watanabe Y, Kinoshita Y, Kamada M, Nodera H, Suzuki H (2010). Mutations of optineurin in amyotrophic lateral sclerosis. Nature.

[18] Yang Y, Hentati A, Deng HX, Dabbagh O, Sasaki T, Hirano M, Hung WY, Ouahchi K, Yan J, Azim AC (2001). The gene encoding alsin, a protein with three guanine-nucleotide exchange factor domains, is mutated in a form of recessive amyotrophic lateral sclerosis. Nat Genet.

[19] Freischmidt A, Wieland T, Richter B, Ruf W, Schaeffer V, Muller K, Marroquin N, Nordin F, Hubers A, Weydt P (2015). Haploinsufficiency of TBK1 causes familial ALS and fronto-temporal dementia. Nat Neurosci.

[20] Nassif M, Woehlbier U, Manque PA (2017). The enigmatic role of C9ORF72 in autophagy. Front Neurosci.

[21] Rudnick ND, Griffey CJ, Guarnieri P, Gerbino V, Wang X, Piersaint JA, Tapia JC, Rich MM, Maniatis T (2017). Distinct roles for motor neuron autophagy early and late in the SOD1(G93A) mouse model of ALS. Proc Natl Acad Sci U S A.

[22] Nassif M, Valenzuela V, Rojas-Rivera D, Vidal R, Matus S, Castillo K, Fuentealba Y, Kroemer G, Levine B, Hetz C (2014). Pathogenic role of BECN1/Beclin 1 in the development of amyotrophic lateral sclerosis. Autophagy.

[23] Valenzuela V, Nassif M, Hetz C (2018). Unraveling the role of motoneuron autophagy in ALS. Autophagy.

[24] Gorlov IP, Gallick GE, Gorlova OY, Amos C, Logothetis CJ (2009). GWAS meets microarray: are the results of genome-wide association studies and gene-expression profiling consistent? Prostate cancer as an example. PLoS One.

[25] Jia P, Ewers JM, Zhao Z (2011). Prioritization of epilepsy associated candidate genes by convergent analysis. PLoS One.

[26] Jia P, Kao CF, Kuo PH, Zhao Z (2011). A comprehensive network and pathway analysis of candidate genes in major depressive disorder. BMC Syst Biol.

[27] Krishnan A, Zhang R, Yao V, Theesfeld CL, Wong AK, Tadych A, Volfovsky N, Packer A (2016). Lash a, Troyanskaya OG: Genome-wide prediction and functional characterization of the genetic basis of autism spectrum disorder. Nat Neurosc.

[28] Maver A, Peterlin B (2011). Positional integratomic approach in identification of genomic candidate regions for Parkinson’s disease. Bioinformatics.

[29] Talwar P, Silla Y, Grover S, Gupta M, Agarwal R, Kushwaha S, Kukreti R (2014). Genomic convergence and network analysis approach to identify candidate genes in Alzheimer’s disease. BMC Genomics.

[30] Behrends C, Sowa ME, Gygi SP, Harper JW (2010). Network organization of the human autophagy system. Nature.

[31] Cronin S, Tomik B, Bradley DG, Slowik A, Hardiman O (2009). Screening for replication of genome-wide SNP associations in sporadic ALS. Eur J Hum Genet.

[32] Cheng X, Ma X, Ding X, Li L, Jiang X, Shen Z, Chen S, Liu W, Gong W, Sun Q (2017). Pacer mediates the function of class III PI3K and HOPS complexes in autophagosome maturation by engaging Stx17. Mol Cell.

[33] Cronin S, Blauw HM, Veldink JH, van Es MA, Ophoff RA, Bradley DG, van den Berg LH, Hardiman O (2008). Analysis of genome-wide copy number variation in Irish and Dutch ALS populations. Hum Mol Genet.

[34] Blauw HM, Al-Chalabi A, Andersen PM, van Vught PW, Diekstra FP, van Es MA, Saris CG, Groen EJ, van Rheenen W, Koppers M (2010). A large genome scan for rare CNVs in amyotrophic lateral sclerosis. Hum Mol Genet.

[35] Blauw HM, Veldink JH, van Es MA, van Vught PW, Saris CG, van der Zwaag B, Franke L, Burbach JP, Wokke JH, Ophoff RA (2008). Copy-number variation in sporadic amyotrophic lateral sclerosis: a genome-wide screen. Lancet Neurol.

[36] Wain LV, Pedroso I, Landers JE, Breen G, Shaw CE, Leigh PN, Brown RH, Tobin MD, Al-Chalabi A (2009). The role of copy number variation in susceptibility to amyotrophic lateral sclerosis: genome-wide association study and comparison with published loci. PLoS One.

[37] Gurney ME, Pu H, Chiu AY, Dal Canto MC, Polchow CY, Alexander DD, Caliendo J, Hentati A, Kwon YW, Deng HX (1994). Motor neuron degeneration in mice that express a human Cu,Zn superoxide dismutase mutation. Science.

[38] Wegorzewska I, Bell S, Cairns NJ, Miller TM, Baloh RH (2009). TDP-43 mutant transgenic mice develop features of ALS and frontotemporal lobar degeneration. Proc Natl Acad Sci U S A.

[39] Herdewyn S, Cirillo C, Van Den Bosch L, Robberecht W, Vanden Berghe P, Van Damme P (2014). Prevention of intestinal obstruction reveals progressive neurodegeneration in mutant TDP-43 (A315T) mice. Mol Neurodegener.

[40] Pattingre S, Tassa A, Qu X, Garuti R, Liang XH, Mizushima N, Packer M, Schneider MD, Levine B (2005). Bcl-2 antiapoptotic proteins inhibit Beclin 1-dependent autophagy. Cell.

[41] Turner BJ, Atkin JD, Farg MA, Zang DW, Rembach A, Lopes EC, Patch JD, Hill AF, Cheema SS (2005). Impaired extracellular secretion of mutant superoxide dismutase 1 associates with neurotoxicity in familial amyotrophic lateral sclerosis. J Neurosci.

[42] Cortez C, Real F, Yoshida N (2016). Lysosome biogenesis/scattering increases host cell susceptibility to invasion by Trypanosoma cruzi metacyclic forms and resistance to tissue culture trypomastigotes. Cell Microbiol.

[43] Wanker EE, Scherzinger E, Heiser V, Sittler A, Eickhoff H, Lehrach H (1999). Membrane filter assay for detection of amyloid-like polyglutamine-containing protein aggregates. Methods Enzymol.

[44] Wang J, Xu G, Borchelt DR (2006). Mapping superoxide dismutase 1 domains of non-native interaction: roles of intra- and intermolecular disulfide bonding in aggregation. J Neurochem.

[45] Hetz C, Thielen P, Matus S, Nassif M, Court F, Kiffin R, Martinez G, Cuervo AM, Brown RH, Glimcher LH (2009). XBP-1 deficiency in the nervous system protects against amyotrophic lateral sclerosis by increasing autophagy. Genes Dev.

[46] Tokuda E, Brannstrom T, Andersen PM, Marklund SL (2016). Low autophagy capacity implicated in motor system vulnerability to mutant superoxide dismutase. Acta Neuropathol Commun.

[47] Huttlin EL, Bruckner RJ, Paulo JA, Cannon JR, Ting L, Baltier K, Colby G, Gebreab F, Gygi MP, Parzen H (2017). Architecture of the human interactome defines protein communities and disease networks. Nature.

[48] Klionsky DJ, Abdelmohsen K, Abe A, Abedin MJ, Abeliovich H, Acevedo Arozena A, Adachi H, Adams CM, Adams PD, Adeli K (2016). Guidelines for the use and interpretation of assays for monitoring autophagy (3rd edition). Autophagy.

[49] Pickford F, Masliah E, Britschgi M, Lucin K, Narasimhan R, Jaeger PA, Small S, Spencer B, Rockenstein E, Levine B (2008). The autophagy-related protein beclin 1 shows reduced expression in early Alzheimer disease and regulates amyloid beta accumulation in mice. J Clin Invest.

[50] Bargsted L, Medinas DB, Martinez Traub F, Rozas P, Munoz N, Nassif M, Jerez C, Catenaccio A, Court FA, Hetz C (2017). Disulfide cross-linked multimers of TDP-43 and spinal motoneuron loss in a TDP-43(A315T) ALS/FTD mouse model. Sci Rep.

[51] Kaplan A, Spiller KJ, Towne C, Kanning KC, Choe GT, Geber A, Akay T, Aebischer P, Henderson CE (2014). Neuronal matrix metalloproteinase-9 is a determinant of selective neurodegeneration. Neuron.

[52] Bosco DA, Morfini G, Karabacak NM, Song Y, Gros-Louis F, Pasinelli P, Goolsby H, Fontaine BA, Lemay N, McKenna-Yasek D (2010). Wild-type and mutant SOD1 share an aberrant conformation and a common pathogenic pathway in ALS. Nat Neurosci.

[53] Forsberg K, Jonsson PA, Andersen PM, Bergemalm D, Graffmo KS, Hultdin M, Jacobsson J, Rosquist R, Marklund SL, Brannstrom T (2010). Novel antibodies reveal inclusions containing non-native SOD1 in sporadic ALS patients. PLoS One.

[54] Medinas DB, Rozas P, Martinez Traub F, Woehlbier U, Brown RH, Bosco DA, Hetz C (2018). Endoplasmic reticulum stress leads to accumulation of wild-type SOD1 aggregates associated with sporadic amyotrophic lateral sclerosis. Proc Natl Acad Sci U S A.

[55] Boers A, Wang R, van Leeuwen RW, Klip HG, de Bock GH, Hollema H, van Criekinge W, de Meyer T, Denil S, van der Zee AGJ (2016). Discovery of new methylation markers to improve screening for cervical intraepithelial neoplasia grade 2/3. Clin Epigenetics.

[56] Huisman C, van der Wijst MG, Schokker M, Blancafort P, Terpstra MM, Kok K, van der Zee AG, Schuuring E, Wisman GB, Rots MG (2016). Re-expression of selected epigenetically silenced candidate tumor suppressor genes in cervical Cancer by TET2-directed demethylation. Mol Ther.

[57] Yang N, Eijsink JJ, Lendvai A, Volders HH, Klip H, Buikema HJ, van Hemel BM, Schuuring E, van der Zee AG, Wisman GB (2009). Methylation markers for CCNA1 and C13ORF18 are strongly associated with high-grade cervical intraepithelial neoplasia and cervical cancer in cervical scrapings. Cancer Epidemiol Biomarkers Prev.

[58] DeJesus-Hernandez M, Mackenzie IR, Boeve BF, Boxer AL, Baker M, Rutherford NJ, Nicholson AM, Finch NA, Flynn H, Adamson J (2011). Expanded GGGGCC hexanucleotide repeat in noncoding region of C9ORF72 causes chromosome 9p-linked FTD and ALS. Neuron.

[59] Morgan S, Shatunov A, Sproviero W, Jones AR, Shoai M, Hughes D, Al Khleifat A, Malaspina A, Morrison KE, Shaw PJ (2017). A comprehensive analysis of rare genetic variation in amyotrophic lateral sclerosis in the UK. Brain.

[60] Mitchell JC, McGoldrick P, Vance C, Hortobagyi T, Sreedharan J, Rogelj B, Tudor EL, Smith BN, Klasen C, Miller CC (2013). Overexpression of human wild-type FUS causes progressive motor neuron degeneration in an age- and dose-dependent fashion. Acta Neuropathol.

[61] Sabatelli M, Moncada A, Conte A, Lattante S, Marangi G, Luigetti M, Lucchini M, Mirabella M, Romano A, Del Grande A (2013). Mutations in the 3′ untranslated region of FUS causing FUS overexpression are associated with amyotrophic lateral sclerosis. Hum Mol Genet.

[62] Gitcho MA, Bigio EH, Mishra M, Johnson N, Weintraub S, Mesulam M, Rademakers R, Chakraverty S, Cruchaga C, Morris JC (2009). TARDBP 3′-UTR variant in autopsy-confirmed frontotemporal lobar degeneration with TDP-43 proteinopathy. Acta Neuropathol.

[63] Pehar M, Harlan BA, Killoy KM, Vargas MR (2017). Role and therapeutic potential of astrocytes in amyotrophic lateral sclerosis. Curr Pharm Des.

[64] Vinsant S, Mansfield C, Jimenez-Moreno R, Del Gaizo MV, Yoshikawa M, Hampton TG, Prevette D, Caress J, Oppenheim RW, Milligan C (2013). Characterization of early pathogenesis in the SOD1(G93A) mouse model of ALS: part II, results and discussion. Brain Behav.

[65] Castillo K, Nassif M, Valenzuela V, Rojas F, Matus S, Mercado G, Court FA, van Zundert B, Hetz C (2013). Trehalose delays the progression of amyotrophic lateral sclerosis by enhancing autophagy in motoneurons. Autophagy.

[66] Settembre C, Di Malta C, Polito VA, Garcia Arencibia M, Vetrini F, Erdin S, Erdin SU, Huynh T, Medina D, Colella P (2011). TFEB links autophagy to lysosomal biogenesis. Science.

[67] Sun Q, Westphal W, Wong KN, Tan I, Zhong Q (2010). Rubicon controls endosome maturation as a Rab7 effector. Proc Natl Acad Sci U S A.

[68] Tabata K, Matsunaga K, Sakane A, Sasaki T, Noda T, Yoshimori T (2010). Rubicon and PLEKHM1 negatively regulate the endocytic/autophagic pathway via a novel Rab7-binding domain. Mol Biol Cell.

[69] Matsunaga K, Saitoh T, Tabata K, Omori H, Satoh T, Kurotori N, Maejima I, Shirahama-Noda K, Ichimura T, Isobe T (2009). Two Beclin 1-binding proteins, Atg14L and Rubicon, reciprocally regulate autophagy at different stages. Nat Cell Biol.

[70] Zhong Y, Wang QJ, Li X, Yan Y, Backer JM, Chait BT, Heintz N, Yue Z (2009). Distinct regulation of autophagic activity by Atg14L and Rubicon associated with Beclin 1-phosphatidylinositol-3-kinase complex. Nat Cell Biol.

[71] Martinez J, Malireddi RK, Lu Q, Cunha LD, Pelletier S, Gingras S, Orchard R, Guan JL, Tan H, Peng J (2015). Molecular characterization of LC3-associated phagocytosis reveals distinct roles for Rubicon, NOX2 and autophagy proteins. Nat Cell Biol.

[72] Wilhelm T, Byrne J, Medina R, Kolundzic E, Geisinger J, Hajduskova M, Tursun B, Richly H (2017). Neuronal inhibition of the autophagy nucleation complex extends life span in post-reproductive C. Elegans. Genes Dev.

[73] Carnio S, LoVerso F, Baraibar MA, Longa E, Khan MM, Maffei M, Reischl M, Canepari M, Loefler S, Kern H (2014). Autophagy impairment in muscle induces neuromuscular junction degeneration and precocious aging. Cell Rep.

[74] Cuervo AM (2008). Autophagy and aging: keeping that old broom working. Trends Genet.

[75] Rubinsztein DC, Marino G, Kroemer G (2011). Autophagy and aging. Cell.

[76] Tanaka Y, Chambers JK, Matsuwaki T, Yamanouchi K, Nishihara M (2014). Possible involvement of lysosomal dysfunction in pathological changes of the brain in aged progranulin-deficient mice. Acta neuropathologica communications.

[77] Xie Y, Zhou B, Lin MY, Wang S, Foust KD, Sheng ZH (2015). Endolysosomal deficits augment mitochondria pathology in spinal motor neurons of asymptomatic fALS mice. Neuron.

[78] Kabuta T, Suzuki Y, Wada K (2006). Degradation of amyotrophic lateral sclerosis-linked mutant Cu,Zn-superoxide dismutase proteins by macroautophagy and the proteasome. J Biol Chem.

[79] Johnson MR, Shkura K, Langley SR, Delahaye-Duriez A, Srivastava P, Hill WD, Rackham OJ, Davies G, Harris SE, Moreno-Moral A (2016). Systems genetics identifies a convergent gene network for cognition and neurodevelopmental disease. Nat Neurosci.

[80] Monti C, Colugnat I, Lopiano L, Chio A, Alberio T. Network analysis identifies disease-specific pathways for Parkinson’s disease. Mol Neurobiol. 2016. 10.1007/s12035-016-0326-0 28004338

