# Supplementary material for: Network approach identifies Pacer as an autophagy protein involved in ALS pathogenesis
Source: Mol Neurodegener. 2019 Mar 27;14:14. doi: 10.1186/s13024-019-0313-9 (PMC6437924; doi:10.1186/s13024-019-0313-9)
Supplement: Supplementary file 1 — Table S1. Genes related to ALS in CNV and HUGE databases. (DOCX 31 kb) [file 13024_2019_313_MOESM1_ESM.docx]

**Table S1.** Genes related to ALS in CNV and HUGE databases.

| **CNV genes** | | | | | | | |
| --- | --- | --- | --- | --- | --- | --- | --- |
| ABHD4 | DEAF1 | HOXB2 | OR4K15 | SPSB3 | ZBTB42 | COL7A1 | WDR16 |
| ADSSL1 | DERA | HOXB3 | OR4K2 | SPSB3 | ZC3H3 | NEK11 | ZNF701 |
| AKT1 | DGKQ | HOXB4 | OR4K5 | SSTR5 | ZCCHC2 | THADA | ZNF83 |
| ALOX15 | DHX35 | HOXB5 | PALM2 | ST8SIA6 | ZFP36 | ZNF684 | SOAT2 |
| ANO9 | DKFZP586H2123 | HOXB6 | PARP16 | STARD13 | ZMYND15 | GJB6 | EVPL |
| ARL8B | DNAH8 | HOXB7 | PBXIP1 | STK39 | ZNF280A | EXOSC9 | SRP68 |
| ARPP-19 | DPP8 | HOXB8 | PDCD11 | STMEM204 | ZNF280B | ZNF137 | ACYP2 |
| ARRB2 | DRD4 | HOXB9 | PDE5A | STRAP | ZUFSP | BTN1A1 | DSEL |
| ARSJ | DRG2 | IDUA | PELP1 | SYN3 | ELMOD2 | COX7C | RARG |
| AVP | EDEM1 | IFT140 | PHRF1 | TAF5 | UCP1 | TNNI2 | C4orf31 |
| AXIN2 | EEF1D | IGFALS | PKP3 | TCERG1 | ATXN1 | C20orf71 | SAR1P3 |
| BAIAP3 | EID2 | IGFALS | PMVK | TELO2 | BTN2A1 | EDA2R | PTPRM |
| BCR | EME2 | INF2 | PPP1R16B | TFG | RIMS2 | RXFP2 | SLCO4C1 |
| BRCA2 | EME2 | IQCD | PRAME | TICAM2 | FAM135B | ARL8B | ZDHHC24 |
| C11orf35 | EPS8 | IRF7 | PRKD1 | TIGD5 | SYK | EDEM1 | BBS1 |
| C12orf52 | EXOC3 | KIAA1370 | ProSAPiP1 | TIMP2 | CCBL1 | BBS7 | ZNF611 |
| C15orf44 | FABP2 | KL | PTPLAD1 | TINP1 | ZER1 | PRDM5 | OR51T1 |
| C16orf38 | FAHD1 | LAMA5 | PUNC | TM4SF5 | TBC1D13 | TRPC3 | OR51F2 |
| C16orf42 | FAM114A1 | LIPC | PYCRL | TMED7 | ENDOG | ITGB7 | OR3A4 |
| C16orf91 | FAM169A | LOC401152 | PYROXD1 | TMEM156 | C9orf114 | CSAD | NP_997216.2 |
| C1QTNF8 | FAM26D | LRRC56 | RAB33B | TMEM175 | GARNL1 | ZNF740 | RGPD6 |
| C20orf151 | FAM83D | LYRM4 | RASSF7 | TNS1 | C14orf177 | CCNA2 | JRKL |
| C8orf73 | FASTKD5 | LZIC | RBP7 | TPCN1 | OR3A1 | N4BP3_HUMAN | LIMS3_HUMAN |
| CABLES2 | FBXL2 | MADD | RDH13 | TPPP | OR3A4 | NOLA2 | RGPD7 |
| CACNA1H | FGFRL1 | MAPK8IP3 | RECQL | TPSAB1 | OR3A2 | RMND5B | Q9Y6V0-3 |
| CALHM2 | FLJ00157 | MAPK8IP3 | RHAG | TPSB2 | LAMA1 | SYT8 | Q86YR2 |
| CALHM3 | FLJ43860 | MED11 | RPS21 | TPSD1 | LRRC30 | CCDC82 | ZNF642 |
| CD83 | GAK | MED13L | RSHL3 | TPSG1 | TEX101 | EPS8 | ZNF600 |
| CDCA2 | GATA5 | MED29 | RWDD1 | TRIM45 | MTMR7 | GEMIN6 | ZNF578 |
| CDH7 | GGTLC2 | MRPS34 | SC16orf103 | TRMT11 | SLC26A6 | PART1 | SERPINB13 |
| CEP72 | GLP1R | MRPS34 | SCT | TTF2 | UQCRC1 | CACNA2D1 | PLUNC |
| CHAC2 | GNAQ | MTMR7 | SERPINB12 | UBE2I | DERA | NAT2 | ZNF808 |
| CLCN7 | GNPTG | MUPCDH | SERPINB13 | UBE4B | STRAP | ADK | 5S_rRNA |
| CLIP2 | GOLGA4 | MYO15A | SERPINB3 | UNKL | DEPDC1B | FMNL2 | Q68DL6 |
| CMTM7 | GOLT1B | N4BP2L1 | SIGIRR | USMG5 | ERCC8 | CHCHD6 | ACTN3 |
| COX7C | GPR128 | N4BP2L2 | SIK1 | USP53 | TRMT11 | DHX57 | OR3A2 |
| CPLX1 | GPR37 | NAPRT1 | SIVA1 | VIM | Q96GK3_HUMAN | SLC12A8 | NUDT16P |
| CR610608 | GSDMD | NDUFC1 | SLC24A1 | VTCN1 | IHPK2 | C1orf176 |  |
| CRAMP1L | GUCY2C | NME3 | SLC26A1 | WBP11 | CDH7 | MAD2L1 |  |
| CRISPLD1 | H2AFJ | NME3 | SLC5A9 | WDR16 | NLP_HUMAN | ANXA5 |  |
| CXCL16 | HAGH | NMNAT1 | SLC9A3 | XCL1 | CDK3 | TMEM155 |  |
| DAD1 | HINT3 | NUBP2 | SLK | XCL2 | HINT3 | ELOVL7 |  |
| DDX54 | HN1L | OR4K1 | SOX8 | YWHAE | NCOA7 | C9orf98 |  |
| **HUGE genes** | | | | | | | |
| SOD1 | VAPB | SHROOM3 | NELL1 | TXNRD1 | HPGD | LPA | PPM1K |
| TARDBP | ITPR2 | ALS2 | NOS3 | MTF1 | HUS1 | LPL | ADRB2 |
| FUS | UNC13A | PVR | NPPA | SPP1 | APOA4 | LTA | ADRB3 |
| PON1 | CHGB | EXOC4 | SLC11A2 | STC1 | APOB | F2 | ZNF519 |
| APOE | OPTN | SCNN1A | OGG1 | TGFB1 | ICAM1 | F5 | DHFR |
| VEGFA | DCTN1 | SELE | ANGPTL4 | ARHGAP18 | APOC3 | F7 | CETP |
| VEGF | CST3 | SUSD1 | PDGFB | GSTO1 | MIF | FGB | MAN1A2 |
| FGGY | KIFAP3 | PPRG | PGF | FIG4 | MMP1 | NLGN1 | CHRNA3 |
| ANG | ALAD | ELP3 | ROBO4 | WNT5A | MMP3 | AGT | CHRNA4 |
| HFE | MAPT | ZFP64 | ACTR5 | WNT10B | MMP12 | AGTR1 | CHRNB4 |
| DPP6 | CHMP2B | DCP1A | C13ORF18 | UPF2 | INHBA | TYMP | MIST |
| GRN | TNF | PRPH | RECK | FBXO8 | ITGA2 | EPO | GSTO2 |
| PON2 | XRCC1 | PSEN1 | CAV1 | GNB3 | ITGB3 | IL18BP | NIPA1 |
| PON3 | SERPINE1 | PTGS2 | STC2 | GSS | LDLR | FMO1 | CP |
| SMN1 | MTHFR | ND5 | VCP | ANK1 | LEP | SUNC1 | C7ORF57 |
| SMN2 | TMEM106B | NEFH | VDR | HGF | LIPC | GALNT1 | MACROD2 |
