# Supplementary material for: Network approach identifies Pacer as an autophagy protein involved in ALS pathogenesis
Source: Mol Neurodegener. 2019 Mar 27;14:14. doi: 10.1186/s13024-019-0313-9 (PMC6437924; doi:10.1186/s13024-019-0313-9)
Supplement: Supplementary file 2 — Table S2. Genes in 12 ALS associated networks. (DOCX 29 kb) [file 13024_2019_313_MOESM2_ESM.docx]

**Table S2.** Genes in 12 ALS associated networks.

| 26s Proteasome | CHMP2B | HDL | MTHFD2L | PPP1R2 | STC2 |
| --- | --- | --- | --- | --- | --- |
| ABLIM | Ck2 | HEPH | MTHFR | PPP1R1A | SUSD1 |
| Actin | Collagen Alpha1 | HFE | MYC | PPP1R9A | TARDBP |
| ACTR5 | Collagen type I | Histone h3 | N-cor | Ppp2c | TCR |
| Adaptor protein 1 | Collagen type IV | Histone h4 | NAA38 | PRNP | TF |
| ADCY | Creb | HNF4A | NADPH oxidase | PRPH | TFR2 |
| AKAP7 | CST3 | Hsp27 | NEFH | PSEN1 | TFRC |
| Akt | Cyclin A | Hsp70 | NELL1 | PSMC3IP | Tgf beta |
| ALAD | Cyclin E | Hsp90 | Nfat (family) | PTGS2 | TNF |
| Alp | Cyclooxygenase | Ige | NFkB (complex) | PVR | Tnf |
| Alpha tubulin | DCP1A | IgG | NfkB-RelA | Rac | TPP2 |
| ALS2 | DCTN1 | IKK (complex) | NfkB1-RelA | Rap1 | TRAF2 |
| Ampa Receptor | dihydrotestosterone | IL1 | NMUR1 | Ras | TRIB1 |
| ANG | DPP6 | IL1/IL6/TNF | Nos | RECK | TRPM7 |
| Angiotensin II receptor type 1 | E2f | IL12 (complex) | NOS3 | RNA polymerase II | Trypsin |
| ANGPTL4 | Elastase | Immunoglobulin | NPPA | ROBO4 | TXNRD1 |
| Ap1 | ELP3 | Insulin | NR4A1 | RTP3 | UBE2H |
| APOE | Endothelin | Integrin | OGG1 | Rxr | Ubiquitin |
| ARHGAP26 | ERBB2 | iron | OPTN | S100 | UNC13A |
| aryldialkylphosphatase | ERK | ISOC2 | P38 MAPK | SCNN1A | USP8 |
| arylesterase | ERK1/2 | ITPR | p85 (pik3r) | SELE | USP54 |
| ATAT1 | Estrogen Receptor | ITPR2 | PAFAH2 | SERPINE1 | VAPB |
| ATF7IP | EXOC4 | Jnk | PARP | SHROOM3 | VCP |
| ATP5C1 | F Actin | KIFAP3 | Pdgf (complex) | SLC11A2 | VDR |
| B2M | Ferritin | Laminin | PDGF BB | SLC25A19 | Vegf |
| B4GALT6 | FGGY | LDL | PDGFB | SLC2A6 | VEGFA |
| BECN1 | Fibrinogen | Lh | peptidase | SLC35A5 | VHL |
| C/ebp | Focal adhesion kinase | LOC729505 | PGF | SLC39A14 | VLDL |
| C13orf18 | FSH | LRP | PHKB | SLIT2 | VPS54 |
| C6orf108 | FUS | MAP2K1/2 | PI3K (complex) | Smad | WNT10A |
| Calcineurin protein(s) | FXC1 | Mapk | Pka | SMN1/SMN2 | XRCC1 |
| Calmodulin | GABPA | MAPK4 | Pkc(s) | Sod | ZBTB11 |
| Calmodulin-CaMKI-Ca2+ | GABPB1 | MAPK15 | Pkg | SOD1 | ZFP36 |
| Calpain | ganglioside GD2 | MAPT | PLC | SP1 | ZFP64 |
| Caspase | GNRH2 | Mek | PLCL1 | SPP1 | ZNF71 |
| CAV1 | GRN | MFHAS1 | PON1 | SQRDL | ZNF318 |
| Cbp/p300 | Growth hormone | MHC Class II (complex) | PON2 | SQSTM1 |  |
| Cbp/p300-Creb | Gsk3 | MINA | PON3 | ST18 |  |
| CCND1 | GUCY | Mmp | PP1-C | STAT |  |
| CHGB | HAMP | MT3 | PP2A | STAT5a/b |  |
| CHMP2 | hCG | MTF1 | PPARG | STC1 |  |
