# Supplementary figures and images for: Network approach identifies Pacer as an autophagy protein involved in ALS pathogenesis
Source: Mol Neurodegener. 2019 Mar 27;14:14. doi: 10.1186/s13024-019-0313-9 (PMC6437924; doi:10.1186/s13024-019-0313-9)

## Slide 1
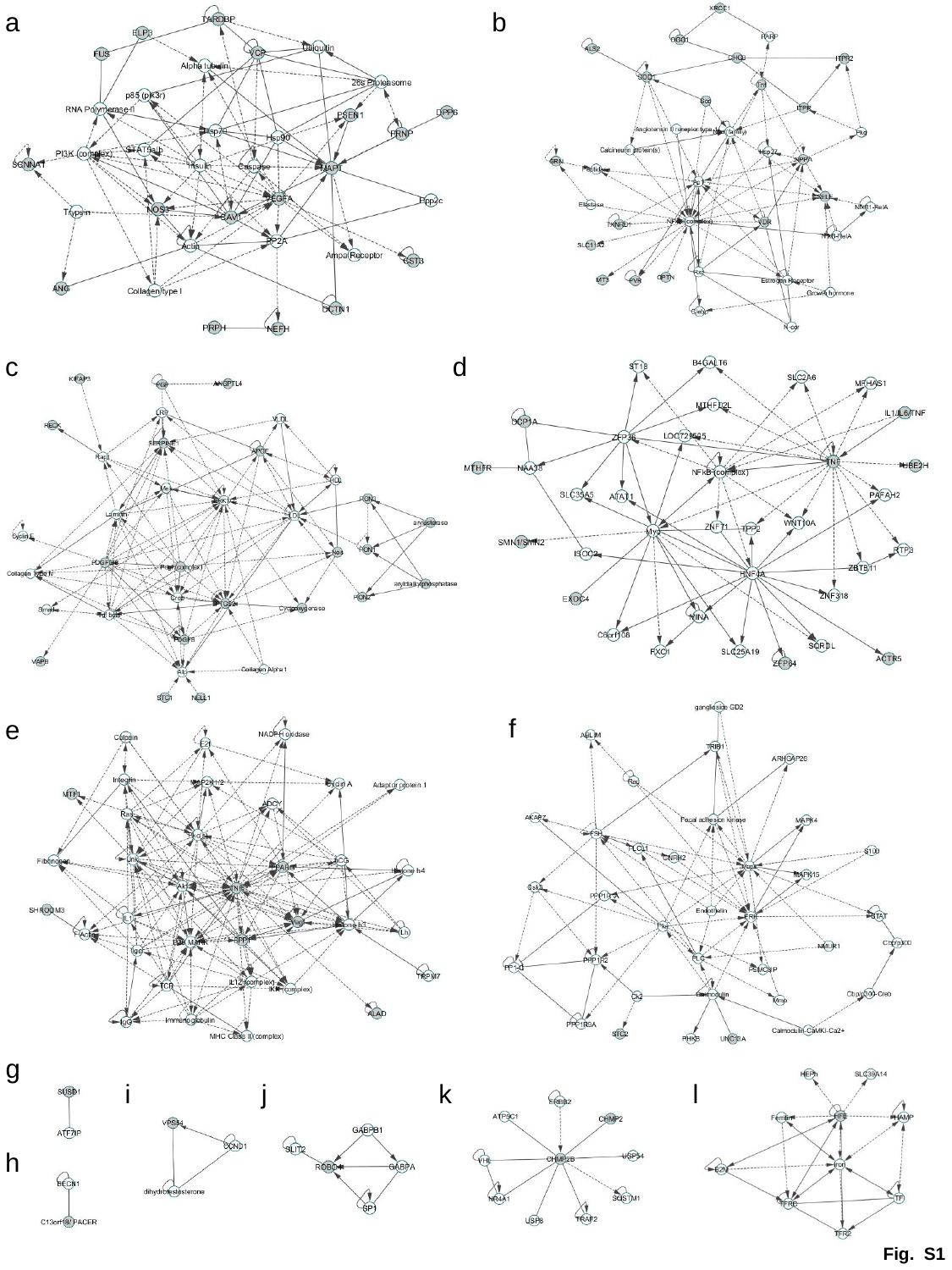

a
b
c
d
f
e
g
i
j
k
l
h
Fig. S1

Supplement: Supplementary file 3 — Figure S1. ALS convergent analysis subnetworks. a-l, ALS disease subnetworks generated by IPA as an output for the convergent analysis approach presented in Fig. 1a. (PPTX 603 kb) [file 13024_2019_313_MOESM3_ESM.pptx]

**Table S3.** Clinical and histopathological data of control and sporadic ALS cases.


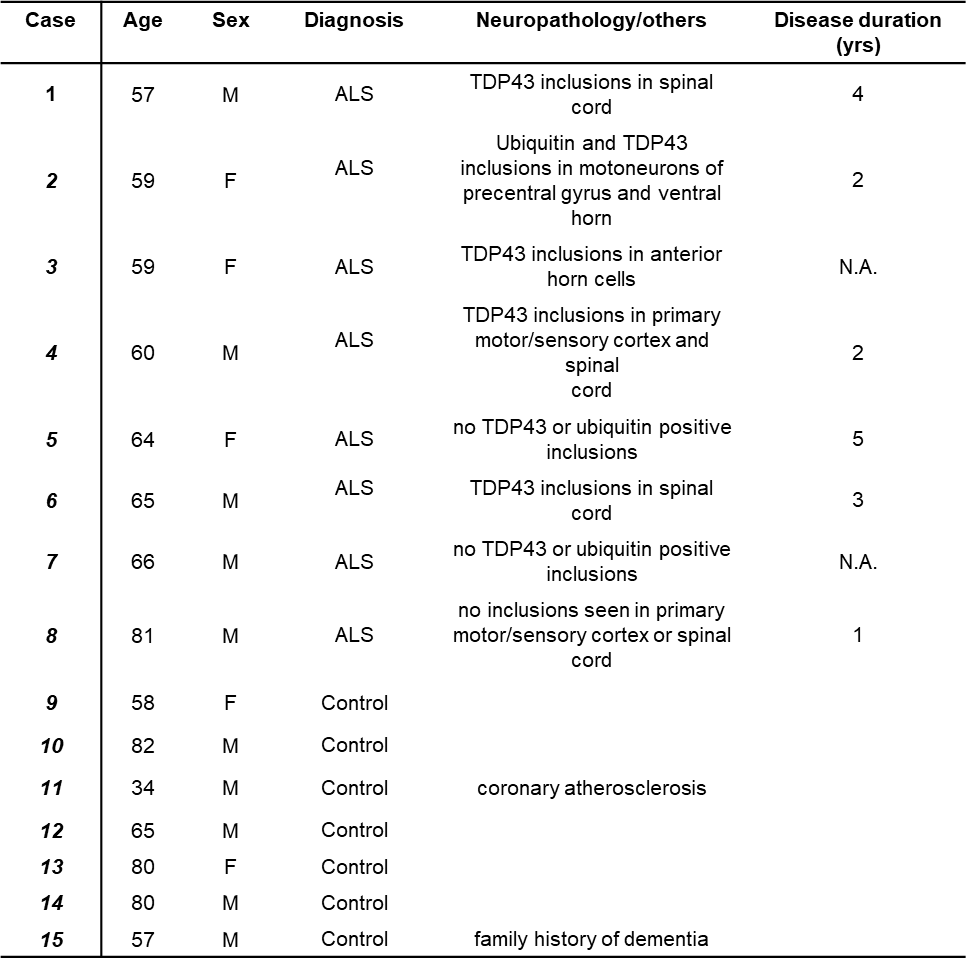

Supplement: Supplementary file 5 — Table S3. Clinical and histopathological data of control and sporadic ALS cases. (DOCX 58 kb) [file 13024_2019_313_MOESM5_ESM.docx]

## Slide 1
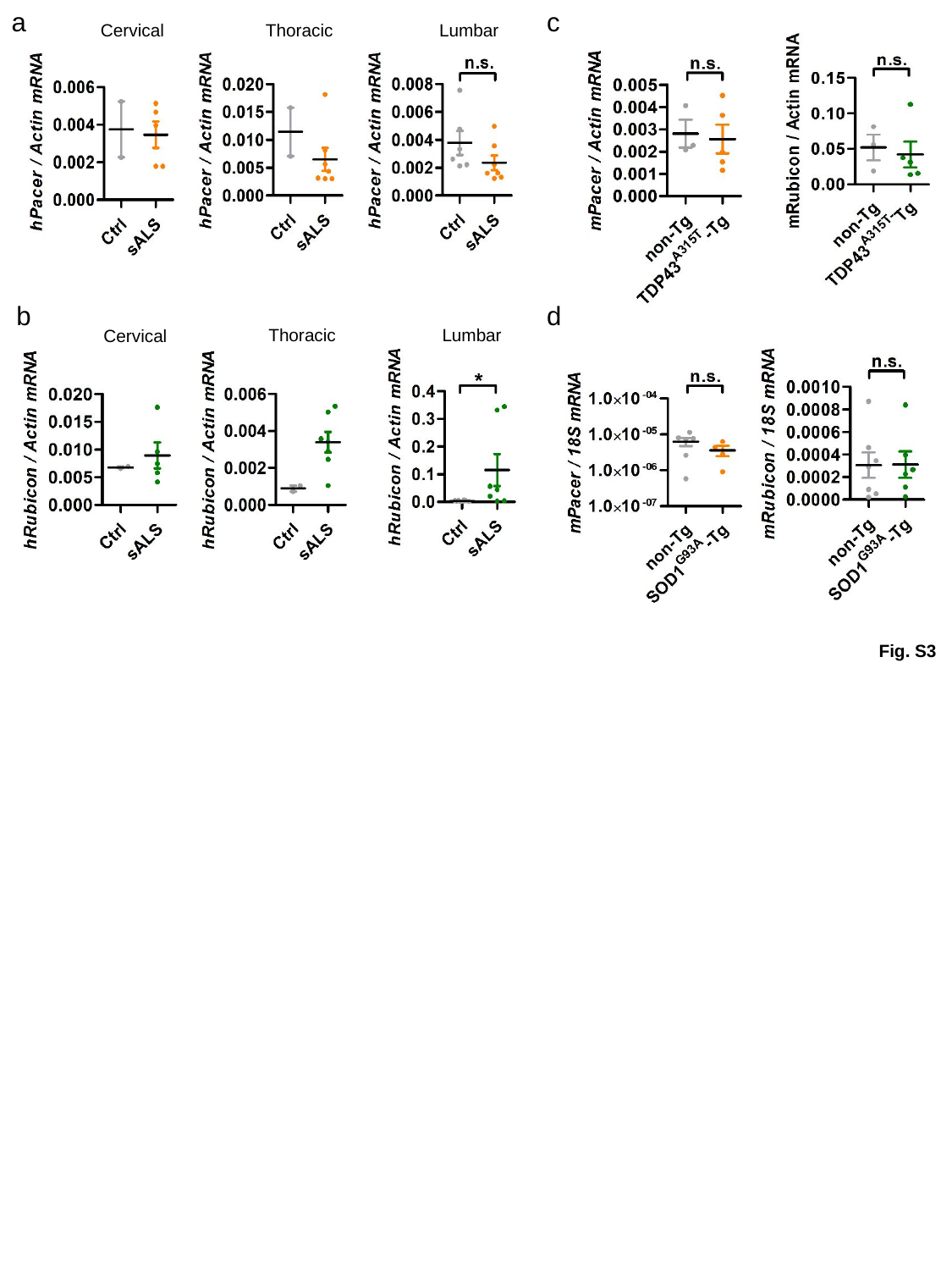

a
c
Lumbar
Thoracic
Cervical
b
d
Lumbar
Thoracic
Cervical
 Fig. S3

Supplement: Supplementary file 6 — Figure S3. Pacer mRNA levels in the lumbar spinal cord from sALS patients and fALS mouse models. a, Human Pacer (hPacer) and b, human Rubicon (hRubicon) mRNA expression was determined by qPCR in postmortem spinal cord sections from sALS patients and age-matched control subjects. Left panel, cervical spinal cord section with Controls n=2 and sALS patients n=6; middle panel, thoracic spinal cord section with Controls n=2 and sALS patients n=7; and right panel, lumbar spinal cord section with Controls n=6 and sALS patients n=7. β-Actin mRNA levels were used for normalization. c, Pacer and Rubicon mRNA expression was determined by qPCR in lumbar spinal cord samples of late symptomatic TDP43A315T transgenic mice (TDP43A315T-Tg, n=5) and their non-transgenic littermate controls (n=3), respectively. β-Actin levels were used for normalization. d, Pacer and Rubicon mRNA expression was determined in the lumbar spinal cord of late symptomatic SOD1G93A transgenic mice (SOD1G93A-Tg) and their non-transgenic littermate controls (both groups, n=7). 18S RNA levels were used for normalization. (PPTX 362 kb) [file 13024_2019_313_MOESM6_ESM.pptx]
