# Supplementary material for: Network approach identifies Pacer as an autophagy protein involved in ALS pathogenesis
Source: Mol Neurodegener. 2019 Mar 27;14:14. doi: 10.1186/s13024-019-0313-9 (PMC6437924; doi:10.1186/s13024-019-0313-9)
Supplement: Supplementary file 4 — Figure S2. Pacer is expressed in neurons in the spinal cord of wild-type mice a, mRNA levels of Pacer in NSC34 cells depleted of Pacer using shRNA constructs (shRNA A and shRNA B) compared to a scrambled control (shCtrl) were determined by real-time. 18s rRNA levels were used for normalization. Statistical analyses were performed using Student’s t-test. Mean, and SEM with only statistically significant p-values are shown: ***, p ≤ 0.001. b, mRNA levels of Pacer, Rubicon and Beclin1 were determined by quantitative PCR in the spinal cord, cortex, hippocampus, cerebellum, muscle, and liver of wild-type C57BL/6 mice (n=8, 4 females, 4 males). mRNA levels in the liver are used as a reference. c, Confocal microscopy of lumbar spinal cord sections of wild-type mice. Z-stack of confocal images, detection of Pacer, the neuron marker NeuN, or the astrocytic marker GFAP, and DAPI detection by immunofluorescence in C57BL/6 46 mice. Scale bars: 300 μm, and 20 μm. Doted inset indicates where higher magnification images were taken. (PPTX 971 kb) [file 13024_2019_313_MOESM4_ESM.pptx]

## Slide 1
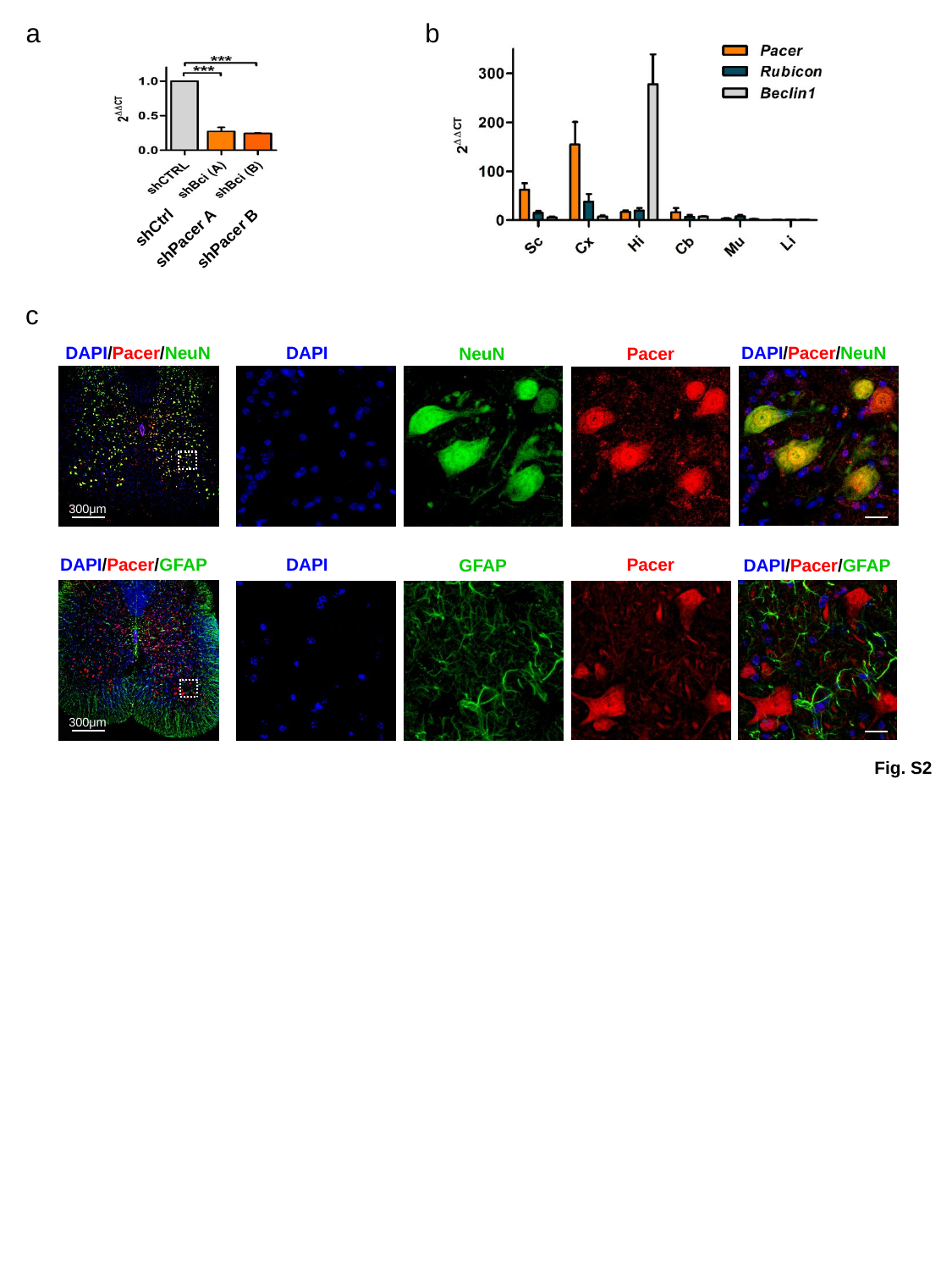

a
b
shCtrl
shPacer A
shPacer B
c
DAPI/Pacer/NeuN
DAPI/Pacer/NeuN
DAPI
Pacer
NeuN
300μm
DAPI/Pacer/GFAP
DAPI
Pacer
GFAP
DAPI/Pacer/GFAP
300μm
300μm
 Fig. S2
