# Supplementary material for: Network approach identifies Pacer as an autophagy protein involved in ALS pathogenesis
Source: Mol Neurodegener. 2019 Mar 27;14:14. doi: 10.1186/s13024-019-0313-9 (PMC6437924; doi:10.1186/s13024-019-0313-9)
Supplement: Supplementary file 7 — Figure S4. Pacer levels and localization in the spinal cord of presymptomatic SOD1G93A transgenic mice. a, Pacer, Rubicon, Beclin1, p62, LC3II protein levels were determined in the lumbar spinal cord of presymptomatic 47 (60 days old) SOD1G93A transgenic mice (SOD1G93A-Tg, n=4) and their non-transgenic littermate controls (n=5). SOD1 human levels are shown as a positive control for SOD1G93A-Tg mice. β-Actin serves as a loading control. Densitometric quantifications of Pacer, Rubicon, Beclin1, p62 and LC3II protein levels normalized to β-Actin levels are shown. b, Confocal microscopy of lumbar spinal cord sections of presymptomatic (60 days old) SOD1G93A transgenic mice (SOD1G93A-Tg, lower panel) compared to age-matched non-transgenic controls (non-Tg, upper panel). Z-stack of confocal images, detection of Pacer, the neuronal marker NeuN in b, or the astrocytic marker GFAP in c. b and c, Nuclei are stained with Hoechst. Scale bar: 30 μm. (PPTX 2790 kb) [file 13024_2019_313_MOESM7_ESM.pptx]

## Slide 1
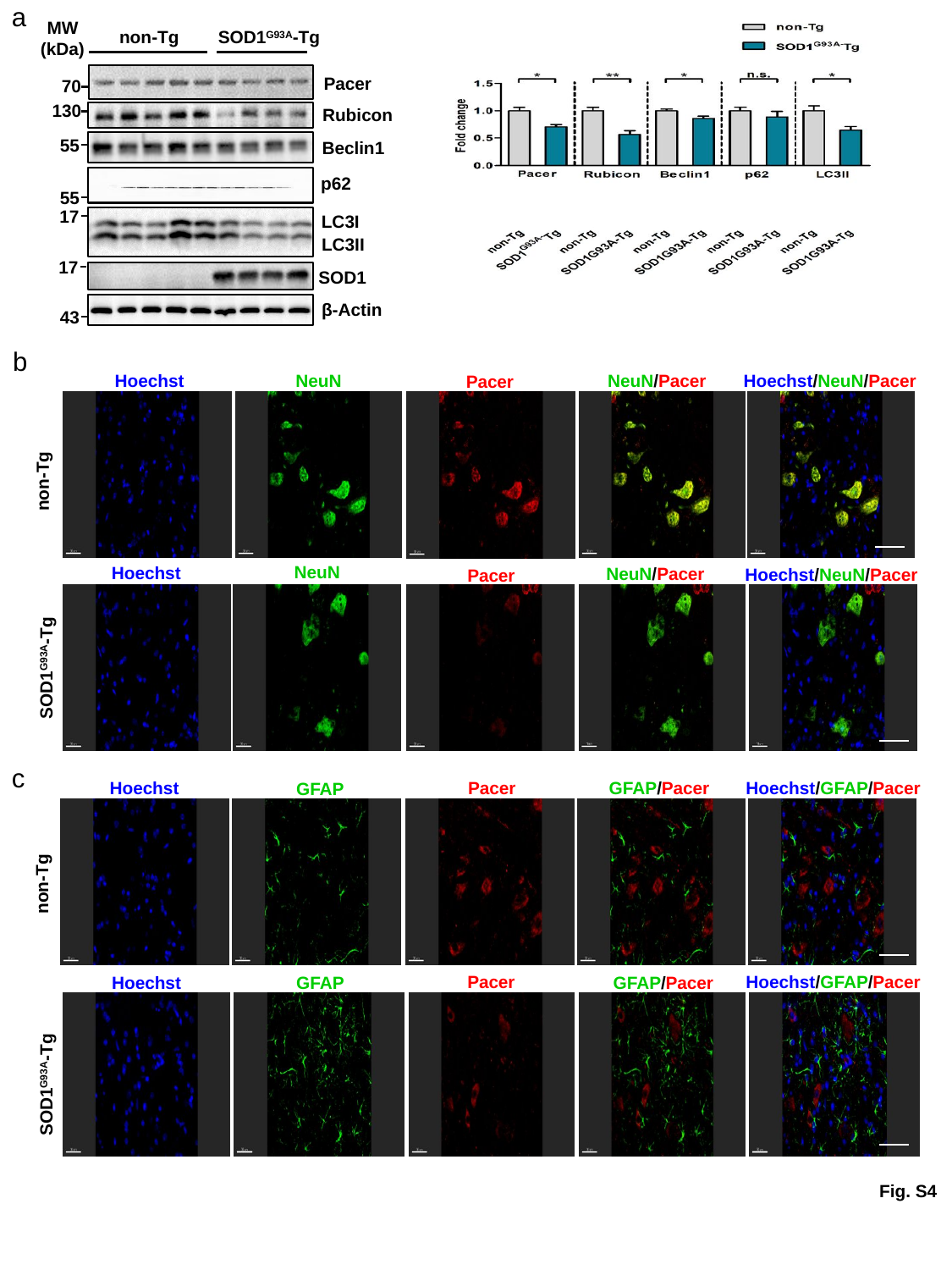

a
MW
(kDa)
non-Tg
SOD1G93A-Tg
Pacer
70
130
Rubicon
55
Beclin1
p62
55
17
LC3I
LC3II
17
SOD1
β-Actin
43
b
Hoechst/NeuN/Pacer
Hoechst
NeuN
NeuN/Pacer
Pacer
non-Tg
NeuN
Hoechst
NeuN/Pacer
Hoechst/NeuN/Pacer
Pacer
SOD1G93A-Tg
c
Hoechst
Pacer
Hoechst/GFAP/Pacer
GFAP/Pacer
GFAP
non-Tg
Hoechst/GFAP/Pacer
Pacer
GFAP
Hoechst
GFAP/Pacer
SOD1G93A-Tg
 Fig. S4
