# Supplementary material for: Network approach identifies Pacer as an autophagy protein involved in ALS pathogenesis
Source: Mol Neurodegener. 2019 Mar 27;14:14. doi: 10.1186/s13024-019-0313-9 (PMC6437924; doi:10.1186/s13024-019-0313-9)
Supplement: Supplementary file 8 — Figure S5. Pacer is expressed in MMP9-positive cells in the presymptomatic spinal cord of SOD1G93A transgenic mice. a, Z-stack confocal images of Pacer with MMP9 in lumbar spinal cord sections of non-transgenic controls (non-Tg, 60 days old) and b, presymptomatic (60 days old) SOD1G93A transgenic mice (SOD1G93A-Tg) at 10X (upper panel, scale bar: 300 μm), 40X (middle panel, scale bar: 30 μm) and 63X (lower panel, scale bar: 15 μm) magnification. Doted insets indicate where higher magnification images were taken. (PPTX 2260 kb) [file 13024_2019_313_MOESM8_ESM.pptx]

## Slide 1
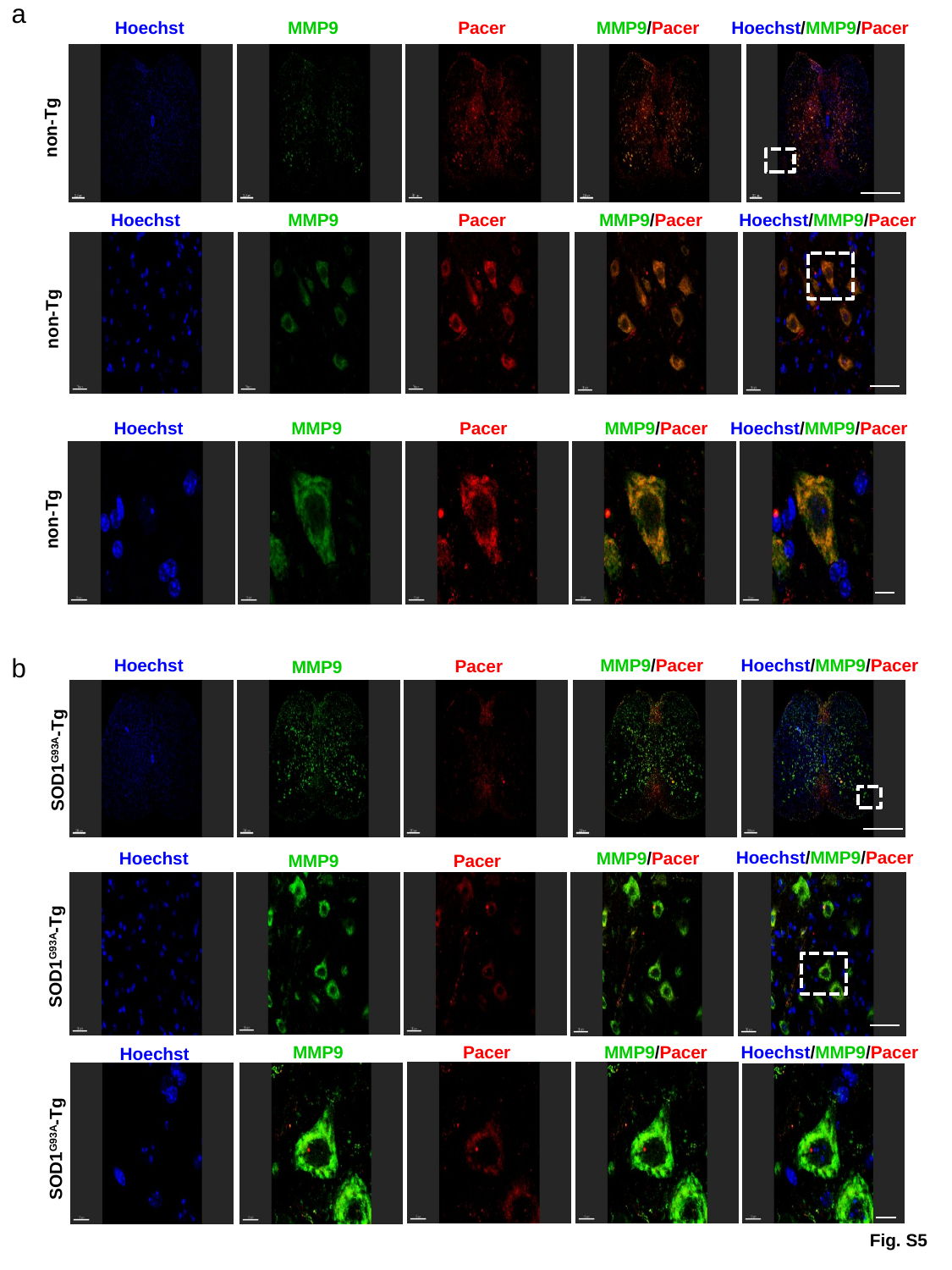

a
MMP9/Pacer
Hoechst/MMP9/Pacer
Hoechst
MMP9
Pacer
non-Tg
Pacer
MMP9/Pacer
Hoechst/MMP9/Pacer
MMP9
Hoechst
non-Tg
MMP9/Pacer
Hoechst/MMP9/Pacer
Hoechst
MMP9
Pacer
non-Tg
b
Hoechst
MMP9/Pacer
Hoechst/MMP9/Pacer
Pacer
MMP9
SOD1G93A-Tg
Hoechst/MMP9/Pacer
Hoechst
MMP9/Pacer
MMP9
Pacer
SOD1G93A-Tg
MMP9
Pacer
MMP9/Pacer
Hoechst/MMP9/Pacer
Hoechst
SOD1G93A-Tg
 Fig. S5
