# Supplementary material for: Network approach identifies Pacer as an autophagy protein involved in ALS pathogenesis
Source: Mol Neurodegener. 2019 Mar 27;14:14. doi: 10.1186/s13024-019-0313-9 (PMC6437924; doi:10.1186/s13024-019-0313-9)
Supplement: Supplementary file 9 — Figure S6. Pacer depletion results in detergent insoluble SOD1 aggregate accumulation. a-b, Densiometric quantification of p62 and Beclin1 levels in the autophagic flux as shown in Fig. 4a. NSC34 cells depleted of Pacer and a scrambled shRNA control (shCtrl) construct were compared (n=3). â-Actin 48 served as a loading control. Statistical analyses were performed using one-way ANOVA and Bonferroni’s post-hoc tests. Mean and SEM are shown. c, NSC34 cells depleted of Pacer were transiently co-transfected with expression vectors for human wild-type or mutant SOD1G93A fused to EGFP. When indicated, human Pacer (hPacer-V5) was co-expressed. NP40-detergent insoluble protein aggregates (NP40 insoluble) were prepared as described in materials and methods. The input is shown as a reference. â-Actin levels in the input serve as a loading control. d-e, Alignment of d shRNA A and e shRNA B to the corresponding target region in mRNA of mouse Pacer and human Pacer. 100% identity between shRNAs A and B to their respective mouse Pacer target sequences. No significant similarity is found to the corresponding region of human Pacer mRNA. (PPTX 2110 kb) [file 13024_2019_313_MOESM9_ESM.pptx]

## Slide 1
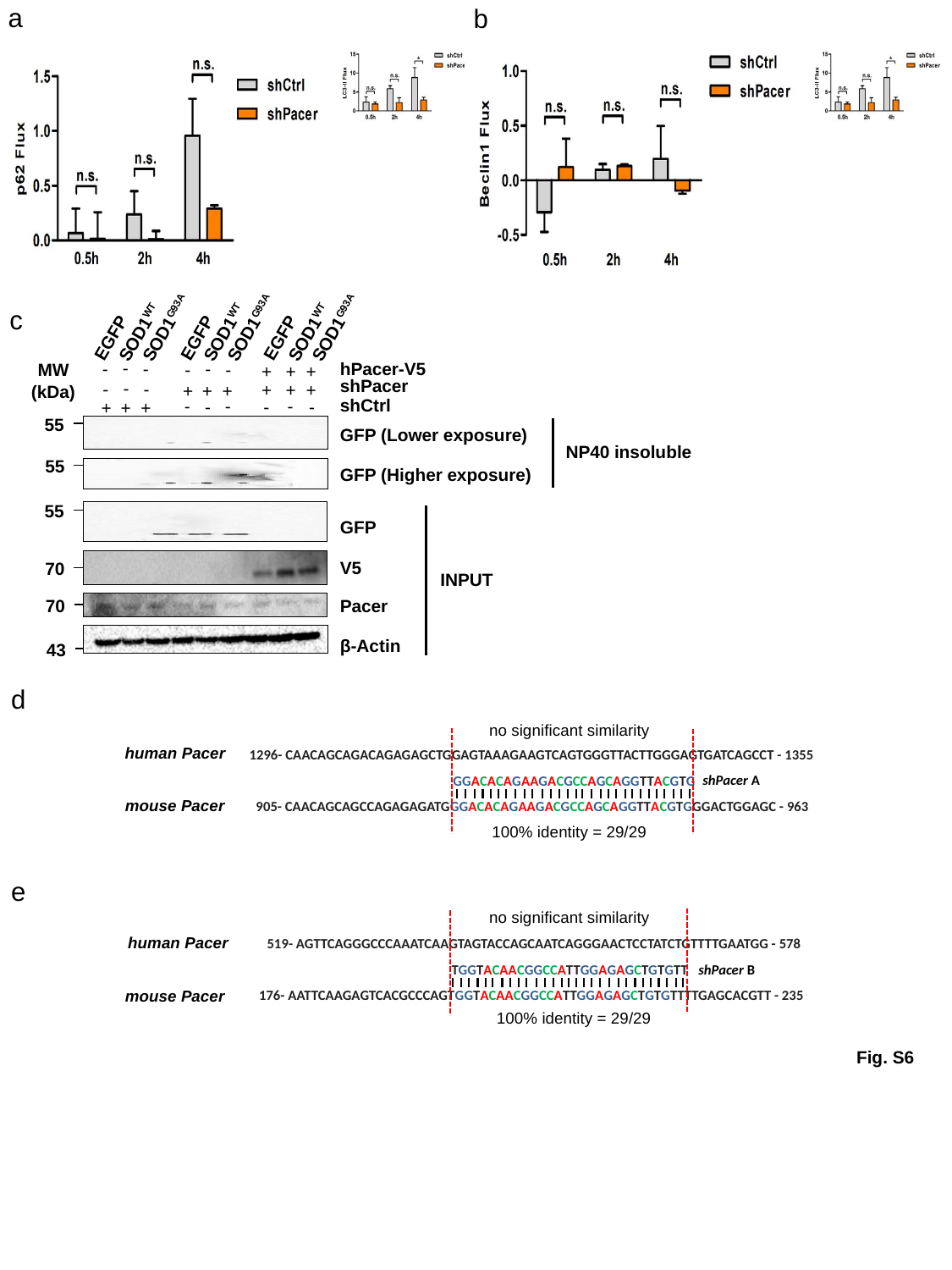

a
b
EGFP
EGFP
EGFP
SOD1G93A
SOD1G93A
SOD1G93A
SOD1WT
SOD1WT
SOD1WT
c
-
-
-
hPacer-V5
-
-
-
MW
(kDa)
+
+
+
shPacer
-
-
-
+
+
+
+
+
+
shCtrl
-
-
-
-
-
-
+
+
+
55
GFP (Lower exposure)
NP40 insoluble
55
GFP (Higher exposure)
55
GFP
V5
70
INPUT
70
Pacer
β-Actin
43
d
no significant similarity
1296- CAACAGCAGACAGAGAGCTGGAGTAAAGAAGTCAGTGGGTTACTTGGGAGTGATCAGCCT - 1355
human Pacer
shPacer A
GGACACAGAAGACGCCAGCAGGTTACGTG
mouse Pacer
905- CAACAGCAGCCAGAGAGATGGGACACAGAAGACGCCAGCAGGTTACGTGGGACTGGAGC - 963
100% identity = 29/29
e
no significant similarity
519- AGTTCAGGGCCCAAATCAAGTAGTACCAGCAATCAGGGAACTCCTATCTGTTTTGAATGG - 578
human Pacer
TGGTACAACGGCCATTGGAGAGCTGTGTT
shPacer B
mouse Pacer
176- AATTCAAGAGTCACGCCCAGTGGTACAACGGCCATTGGAGAGCTGTGTTTTGAGCACGTT - 235
100% identity = 29/29
Fig. S6
