# Supplementary material for: Network approach identifies Pacer as an autophagy protein involved in ALS pathogenesis
Source: Mol Neurodegener. 2019 Mar 27;14:14. doi: 10.1186/s13024-019-0313-9 (PMC6437924; doi:10.1186/s13024-019-0313-9)
Supplement: Supplementary file 10 — Figure S7. Depletion of Pacer leads to SOD1 aggregate accumulation. a, NSC34 cells were transiently transfected with constructs for shCtrl, shPacer, EGFP, SOD1WT-EGFP and SOD1G93A-EGFP, inclusions are shown with white arrows (Representative images of 3 independents experiments). Scale bar 40 μM. b-c, Stable NSC34 cell lines expressing b shPacer and c shRubicon were established. Knockdown was confirmed by Western blot. HSP90 and b-Actin were used as loading controls, respectively. d, Percentage of cell death at 48 h (SytoxBlue positive, SB+) in NSC34 stable lines expressing shPacer, shRubicon, and shCtrl constructs. Cells were transiently transfected with plasmids for EGFP or SOD1G93A-EGFP. In d statistical analyses were performed using one- 49 way ANOVA and Bonferroni’s post-hoc tests. Mean and SEM with only statistically significant p-values are shown: *, p ≤ 0.05; and **, p ≤ 0.01. (PPTX 777 kb) [file 13024_2019_313_MOESM10_ESM.pptx]

## Slide 1
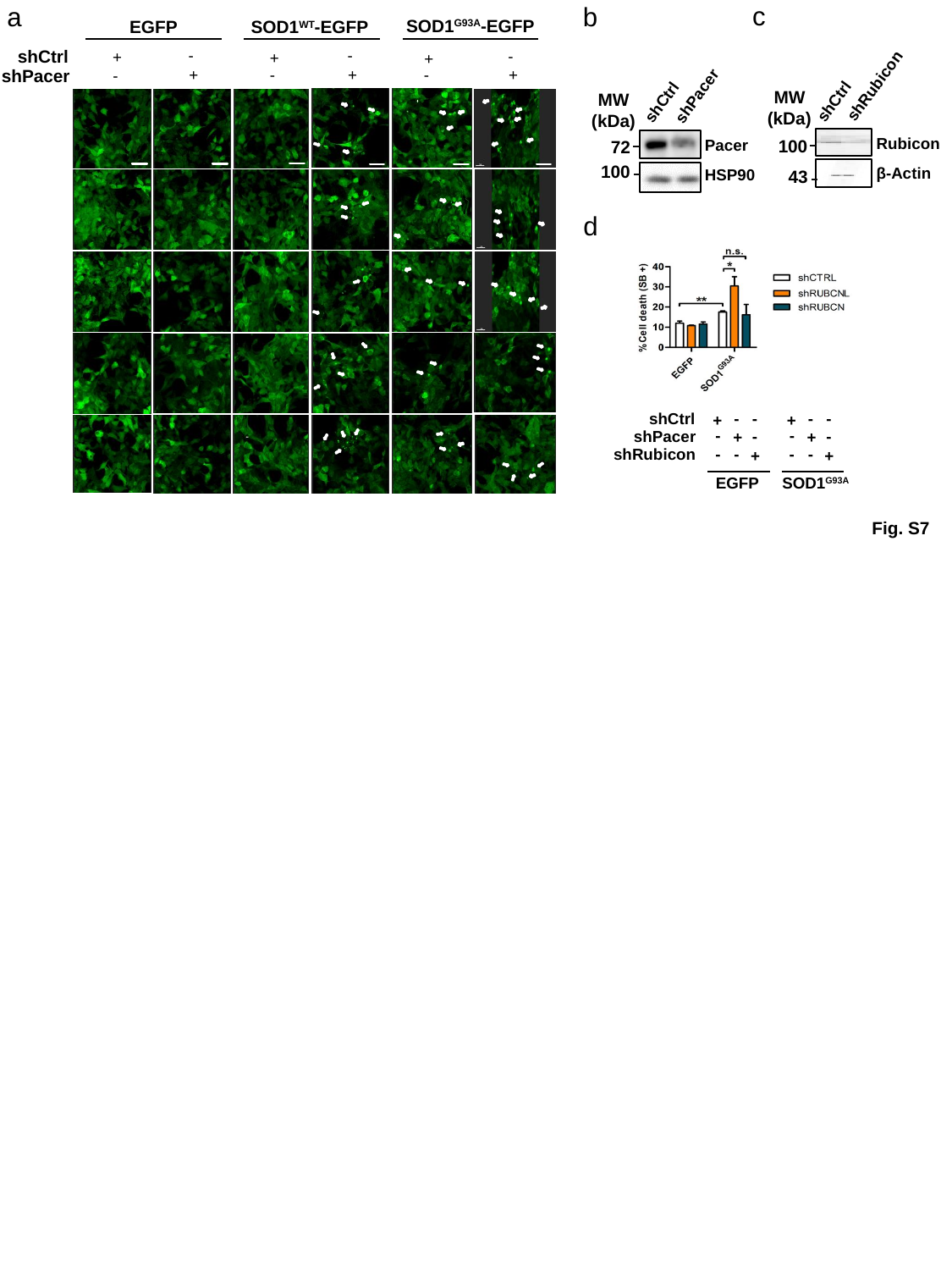

c
b
shRubicon
shCtrl
shCtrl
shPacer
MW
(kDa)
MW
(kDa)
Rubicon
Pacer
100
72
100
β-Actin
HSP90
43
a
SOD1G93A-EGFP
EGFP
SOD1WT-EGFP
shCtrl
-
-
-
+
+
+
-
+
-
+
shPacer
+
-
d
-
-
-
-
shCtrl
+
+
-
-
-
-
shPacer
+
+
shRubicon
-
-
-
-
+
+
EGFP
SOD1G93A
Fig. S7
